# A Systematic Review of Music Therapy Practice and Outcomes with Acute Adult Psychiatric In-Patients

**DOI:** 10.1371/journal.pone.0070252

**Published:** 2013-08-02

**Authors:** Catherine Carr, Helen Odell-Miller, Stefan Priebe

**Affiliations:** 1 Unit for Social and Community Psychiatry, Barts and the London School of Medicine and Dentistry, Queen Mary University of London, London, United Kingdom; 2 Music and Performing Arts Department, Anglia Ruskin University, Cambridge, United Kingdom; Maastricht University Medical Centre, The Netherlands

## Abstract

**Background and Objectives:**

There is an emerging evidence base for the use of music therapy in the treatment of severe mental illness. Whilst different models of music therapy have been developed in mental health care, none have specifically accounted for the features and context of acute in-patient settings. This review aimed to identify how music therapy is provided for acute adult psychiatric in-patients and what outcomes have been reported.

**Review Methods:**

A systematic review using medical, psychological and music therapy databases. Papers describing music therapy with acute adult psychiatric in-patients were included. Analysis utilised narrative synthesis.

**Results:**

98 papers were identified, of which 35 reported research findings. Open group work and active music making for nonverbal expression alongside verbal reflection was emphasised. Aims were engagement, communication and interpersonal relationships focusing upon immediate areas of need rather than longer term insight. The short stay, patient diversity and institutional structure influenced delivery and resulted in a focus on single sessions, high session frequency, more therapist direction, flexible use of musical activities, predictable musical structures, and clear realistic goals. Outcome studies suggested effectiveness in addressing a range of symptoms, but were limited by methodological shortcomings and small sample sizes. Studies with significant positive effects all used active musical participation with a degree of structure and were delivered in four or more sessions.

**Conclusions:**

No single clearly defined model exists for music therapy with adults in acute psychiatric in-patient settings, and described models are not conclusive. Greater frequency of therapy, active structured music making with verbal discussion, consistency of contact and boundaries, an emphasis on building a therapeutic relationship and building patient resources may be of particular importance. Further research is required to develop specific music therapy models for this patient group that can be tested in experimental studies.

## Introduction

Acute in-patient care is offered when a patient is in severe crisis to provide a “safe and therapeutic setting for service users in the most acute and vulnerable stage of their illness” [1]. Admissions may be voluntary or through compulsory legal detention. Reasons for admission may be for assessment, treatment of acute symptoms or relapse prevention with the aim for patients to recover to a point where they are able to return to the community. Length of admission varies, however within the United Kingdom (UK), it has reduced to an average of less than 4 weeks [2], and is continuing to decrease.

Whilst the evidence base for music therapy in the treatment of serious mental disorders is growing [3]-[7], little attention has been paid to the delivery of music therapy in acute in-patient treatment. Research to date suggests many more sessions are required for clinically meaningful effects than may be accessed in hospital [4] and there has been little distinction between interventions offered in acute stages of illness, and those offered long-term [8], [9]. A number of models and methods of music therapy have developed in mental health care, yet specific approaches to account for the acute in-patient context have not been systematically examined [10]-[14]. Against this background, we conducted a systematic review addressing the following questions:

1. What are the clinical aims and considerations for music therapy with acute adult psychiatric patients in acute hospital settings?
2. How is music therapy provided in these settings in terms of frequency, duration and methods used?
3. What are the findings from outcome studies conducted in these settings?

## Methods

A systematic review was conducted utilising narrative synthesis [15]-[17]. Methods were specified in advance in a protocol [Supporting information S1].

### Eligibility Criteria

#### Definition of intervention

Music therapy is a systematic intervention that uses music experiences and the relationships that develop through these to promote health [18]. Music may be actively produced by patient and therapist (for example, improvisation on musical instruments), or receptive, such as listening to pre-recorded music. The type of musical interaction, level of structure and amount of verbal discussion may vary depending upon the music therapist’s approach, client characteristics and diagnosis. Interventions can take the form of group or individual therapy and aims will vary according to the specific needs of the patient.

#### Criteria

Papers were included if they described music therapy as the main component of treatment with adult in-patients (ages 18+) admitted for treatment of acute symptoms in psychiatric hospitals. Interventions used active and/or receptive musical activities as the primary treatment component in conjunction with the relationships formed through these activities to promote health [18]. Papers were excluded if a) music was not the primary focus of the intervention, for example, dance movement psychotherapy might use music within the intervention, but the focus is upon the physical use of body and movement; b) music was provided without a focus upon relationships, for example use of background music to alter the surrounding environment, music for private listening without therapist involvement, or provision of instruments for patients to access in their own time on the ward; c) the primary aim of the intervention was not to promote health, for example, music lessons with the aim of increasing musical knowledge or skill. Interventions delivered by non-music therapists were included if the intervention met the above criteria.

Papers describing both in-patient and out-patient treatment were included but only features of in-patient work were extracted. Papers focusing upon patients with an organic mental illness (ICD F00-09) were excluded. Data on diagnosis-specific and general symptoms, motivation, attendance, musical engagement, musical preference, social and behavioural changes were extracted. There were no restrictions on study design, publication year or language.

### Information Sources and Search Strategy

Databases were identified and searched based on existing guidance and reviews [4], [19], [20]. Relevant journals, library catalogues and conference proceedings were then hand-searched. The full database and journal list can be found in the supporting information [Supporting information S2]. References were inspected for further relevant literature, and a forward citation search performed using ISI Web of Science. The search was repeated after 10 months and completed on 30^th^ March 2012.

The following search terms were employed:

[* musi* or musi* or * sound* or sound* or * acou* or acou* or gim^1^ in title, abstract, index terms of REFERENCE] or [music* in interventions of STUDY] and [psychiatr* or mental* or schizophreni* or psychosis or psychotic].

The search term ‘gim’ was included to find papers relating to Guided Imagery in Music – a specific approach used by music therapists involving receptive listening with the therapist guiding the patient through images evoked.

### Study Selection

Detailed citations (title and abstract) were screened by the author (CC) and marked as include; exclude or uncertain. Full papers were retrieved and those marked as uncertain were reviewed against the inclusion criteria. Five authors were contacted for further information. All responded, and three provided references to a further five papers. Searches were managed and saved using Reference Manager (v.12, Thomson Reuters).

### Data Extraction

Details of research design and method, country, diagnosis, group/individual, frequency, length, number of sessions offered and attended, duration of therapy, music therapy approaches and techniques, theories informing rationale, client and setting specific features, reported experiences and prospective study results were entered into an excel spreadsheet which was then imported into NVivo (v.18, QSR International) for qualitative analysis [Supporting information S3]. For clinical outcome studies, sample size, mean scores and standard deviations for each time point were extracted along with statistical tests of significance. Twenty-five percent of the included papers were checked for accuracy of inclusion, coding and quality assessment by a second researcher (SO). Third and fourth researchers (SP and HO-M) were available for further discussion and resolution.

### Assessment of Risk of Bias

As this review combined clinical, theoretical and research papers, the EPPI “weight of the evidence” (WoE) approach was employed [21], [22]. In this approach, papers are rated not only on their methodological quality (WoEA), but also on the relevance of the study design to the review question (WoEB) and overall relevance to the review question as a whole (WoEC). These ratings are combined to provide an overall “weight of the evidence”(WoED). For research methodology (WoEA), Downs & Black’s [23] checklist was selected for prospective quantitative studies.

For qualitative studies, the “Quality Framework” [24] was used by scoring each area as either present (1) or absent (0). Finally, for practitioner based papers (such as expert opinion or clinical theoretical papers), guidelines from the Social Care Institute for Excellence were employed [25]. Scores were averaged to make an overall score (WoED) and classified as Low (0–0.35), Medium (0.36–0.69) or High (0.7-1). Any papers with a low overall (WoED) or methodological score (WoEA) are reported in the results but were excluded from all analyses. To examine publication and selective reporting bias, study protocols and outcomes reported in the method were compared with published results.

### Synthesis

Synthesis used elements and tools from guidance for the narrative synthesis of mixed types of data and followed three stages of 1. Developing a preliminary synthesis, 2. Exploring relationships within and between studies and 3. Assessing the robustness of the synthesis [15]-[17]. Preliminary synthesis (step 1) for objectives 1 and 2 employed tools of thematic synthesis and vote counting of themes within papers [26]. Papers were coded line by line for each area of extraction and grouped thematically. A thematic framework was tabulated and organized by sub-groups of country, approaches, interventions, research design and outcomes. This was then developed into a conceptual map of ‘analytical themes’ to synthesize setting-specific features and approaches [26]. Clinical aims, modifications to practice and reasons for this were grouped into similar features. For objective 3, experimental group pre-post differences and differences between groups post-intervention were converted to standardised mean differences and tabulated.

Relationships between studies (step 2) were explored using tabulation and vote counting. In the thematic analysis, similar features specific to acute inpatient work were grouped together, along with their impact upon therapy and approaches taken to address them. Vote counting was used to check coverage of themes and these were stratified by year of publication, country and length of stay to explore any potential patterns or influences. Experimental results were tabulated and grouped by outcome. Vote counting was used to rank outcomes according to the size and direction of standardized mean differences and statistical significance. Outcomes were then compared by intervention, number of sessions received and study quality.

Robustness of the synthesis product (step 3) was assessed through quality assessment, Doctoral supervision with a music therapist (HO-M) and psychiatrist (SP) and presentations to a mental health research group consisting of Psychologists and Psychiatrists within the authors’ institution and to music therapists at an international music therapy conference.

## Results

**Figure 1:**
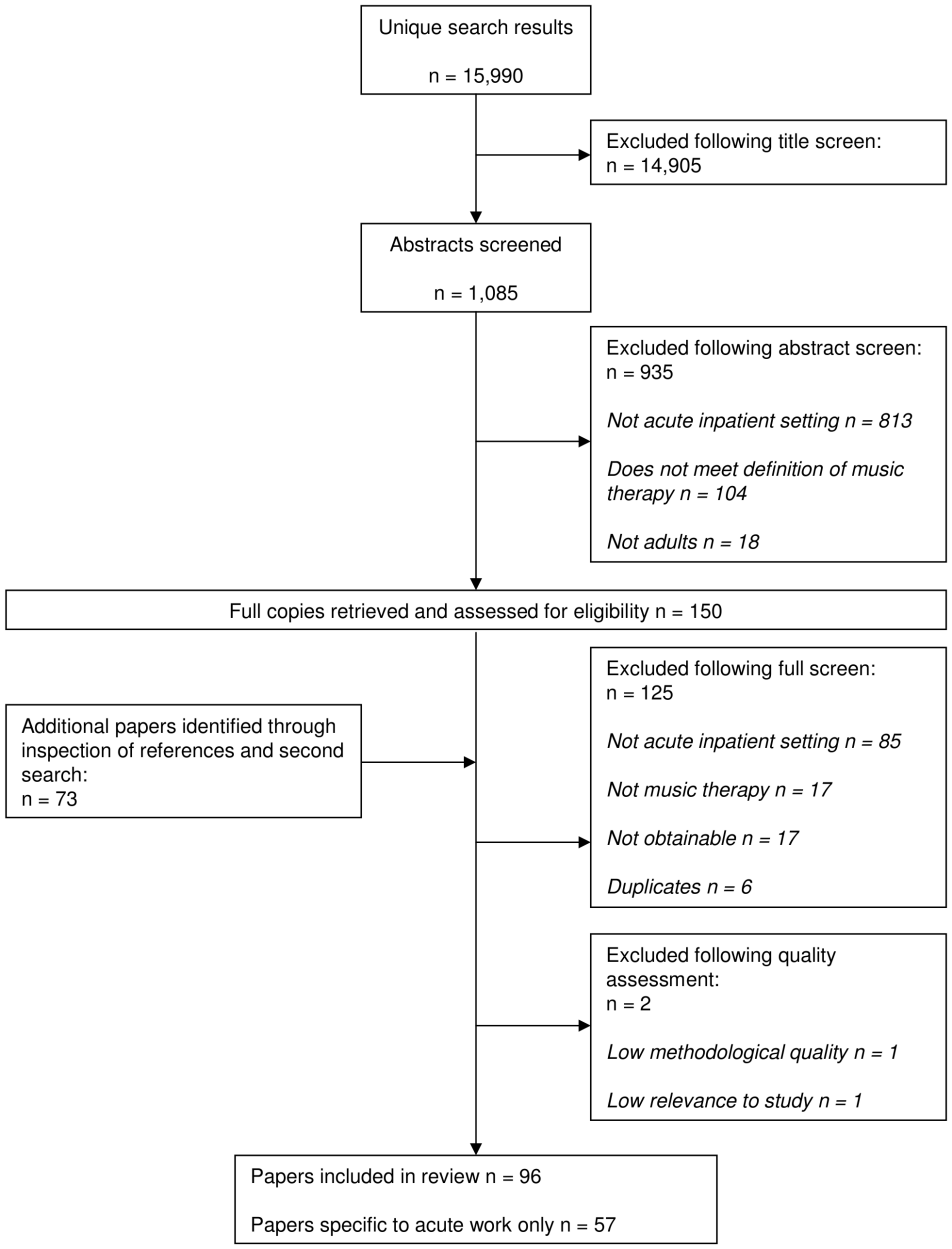
QUORUM Diagram.

Ninety-eight papers [4], [10], [12], [13], [27]-[120], were identified for inclusion in the review [PRISMA diagram, Figure 1]. Of these, 57 covered acute work specifically, whilst 41 included acute work as part of a wider discussion of practice in mental health. The majority of papers came from the USA (N = 32) and UK (N = 17) and were clinical theoretical discussions or case studies (N = 63), whilst research and service evaluations comprised 35 of the included papers. Two papers were rated as low quality and therefore excluded from the main thematic analysis [42], [92]. Paper characteristics are shown in the supporting information [Supporting information S4].

### Thematic Synthesis

#### Clinical Aims

Clinical aims were conceptualized as building of interpersonal relationships, self-expression and personal resources [Table S5]. Immediate and short-term aims were most prominent, with priority given to establishing the therapeutic engagement of patients. Initial engagement aims related specifically to making contact and building a therapeutic relationship, active involvement of patients in either activity or therapy (both as in and out-patient) (N = 34) and fostering motivation and volition (N = 22). Immediate aims focused upon the reduction of anxiety, management of emotional arousal, building internal and external organization and providing reality orientation. Once engagement was established, goals then focused upon short term features to address the patient’s immediate situation within hospital. These included work on coping skills (N = 21), building musical resources, defences and boundaries (N = 39), prevention of relapse and exploration of issues that led to hospitalization (N = 13). Interpersonal processes focused upon making nonverbal contact with others, building awareness of how one interacts with others, building and improving relationships, teamwork and socialisation. Related to this was communication (N = 50) where aims focused upon encouragement of nonverbal expression, self-expression and verbal communication with others. Emotional aims (N = 47) focused upon management of arousal, self-expression and building awareness and naming of affective states whilst cognitive aims (N = 33) focused upon sustaining and increasing attention and organizing patients’ physical actions, behaviours and thoughts. Papers from the USA, UK and Denmark noted setting specific aims of helping patients to deal with hospitalization, such as decreasing hospital and discharge anxiety (N = 24), and changing the atmosphere on the ward, such as improving patient staff integration (N = 16).

Aims relating to symptoms were less frequently mentioned (N = 20). They focused upon reality orientation in psychosis and the reduction of depression and anxiety. Papers varied in opinion regarding the extent to which music therapy should aim to address specific symptoms and problems directly. Three papers stated specifically that this was not a goal of therapy [30], [65], [106]. Both Solli [65] and Leite [106] suggested that complete elimination of symptoms or problems may be unrealistic given the short time-frame of work and therefore suggest building of patient strengths and resources to help them cope with their current situation. Solli & Rolvsjord [110] suggested four features of music therapy that might assist in addressing symptoms of psychosis: motivation, structure, emotional expression and social participation. Nine papers, all informed by Yalom’s model of in-patient therapy [122], proposed aims oriented towards supporting and reinforcing strengths and skills rather than longer term insight [13], [49], [76], [79], [107]-[109], [118], [119]. Mössler et al. [76] linked this to Storz’s [112] ‘potential orientation’ and ‘resource orientation’ found in other short term psychotherapies. Similarly psychodynamic and psychoanalytically informed approaches focused upon building and strengthening defence structures [52], [65], [119], aiming at “containing action and delineating boundaries” [65] rather than opening up of emotions or deep connection with others.

#### Characteristics of Delivery

Service characteristics are shown in Table 1, and content and structure of sessions in Table 2. Music therapy was offered to patients with a range of diagnoses. Twenty-six papers focused upon specific diagnoses, usually schizophrenia or psychosis, 13 of which were individual case studies and 8 were for research or service evaluation.

**Table 1:** Delivery of music therapy across included papers.

**Number of papers (N)**	All	98
	Acute only	57
**Mixed Diagnoses (N)**		72
**N Sessions attended (range)**		1–133
**Link to outpatient work (N)**		22
**Duration in-patient stay (range, weeks)**		0.6–75
**Duration of therapy (range, weeks)**	All	0.2–129
	Acute only	0.2–38
**Location (N)**	On ward	23
	Off ward	11
	Both	8
**Type of therapy (N)**	Individual	17
	Group	45
	Both individual and group	29
**Individual work**	Frequency per week	1–6
	Length of session (minutes)	10–60
**Group work**	Frequency per week	0.5–6
	Length of session (minutes)	30–90
	Group size (range)	3–40
	Co-work with another member of staff	14
**Group Format (N)**	Open	23
	Semi-open	10
	Semi-closed	3
	Closed	6
	Both open and closed	7

**Table 2:** Session structure and content across included papers.

			N Papers
**Direction**	**Therapist**	Directive	44
		Non-directive	51
	**Session**	Therapist led	36
		Patient Led	53
**Structure**		Opening and closing events	30
		High structure	28
		Flexible structure	40
		Low structure	11
**Active techniques**	**Improvisation**	Free	50
		Structured	27
		Thematic	19
		Playback of recording	12
	**Composing**	Music composition	2
		Songwriting	21
	**Playing pre-composed music**	Ensemble playing	21
		Singing	33
		Rhythmic playing	7
	**Didactic/tuition**		11
**Receptive techniques**	**Listening**	Live reception	6
		For relaxation	12
		Music selection & discussion	18
		Structured affective listening	12
		Reminiscence	-
		Guided Imagery in Music	10
	**Music based activity**	Lyric Analysis	10
		Music collage	3
		Music games	8
**Use of other arts modalities**		Movement	12
		Other arts forms	17
**Use of verbal reflection**			63

The duration of in-patient stay ranged from 3 days to 75 weeks. Duration of therapy upon acute wards ranged from a single session to 38 weeks. The mode frequency of therapy was twice a week, and ranged from fortnightly to 6 sessions per week. Therapists working in hospitals with a short length of stay tended to offer a greater frequency of sessions, particularly in the USA. Open ward groups were the predominant form of delivery. Smaller semi-open or closed groups were run to meet specific needs or levels of functioning. Group and individual work was also combined (N = 29), whilst other reports focused on individual work only (N = 17) or included outpatient work (N = 22).

Features impacting upon the delivery of music therapy can be found in supporting information S5 and S6 [Supporting information S5] [Supporting information S6]. Setting characteristics included the institutional model and structure, communication with the multi-disciplinary team, ward environment, high patient turnover and shorter time-frame to work. The diversity of patients in terms of symptom severity, functioning levels, reaction to hospitalization, previous therapy experiences, and motivation to enter into therapy similarly impacted upon therapy delivery.

The integration of music therapy within the models and systems of the institutional setting meant prioritization of multidisciplinary team communication, provision of clear information to both patients and staff, and provision of a programme to maximize patient access and staff support [34], [40], [75], [91], [104]. Solli [108] suggests therapists tailor their work at different individual, group, ward and hospital levels. Work may also extend to links with the community through sessions accessible to outpatients eg. [48], [86] or direction of patients to community resources eg. [13], [48], [64], [108], [109], although barriers such as continuity of service and ability to follow-up patients were identified [40], [116].

Attendance and engagement were key challenges due to symptom severity, high patient turnover and short lengths of stay. Low attendance was generally experienced negatively by patients [50], [91] and impacted upon the group dynamic [117]. Access to sessions was limited by mental state, external events or by institutional barriers such as the time taken for referral and assessment and ongoing demands upon the patient whilst in hospital [34], [52]. Talwar et al. [116] also observed that uptake of outpatient attendance was rare unless several in-patient sessions were attended.

Engagement of patients in therapy itself was noted as a difficult process either due to anxiety in use of the medium [29], [39], [59], [62], [65], [76], [82], [85], [90], [95], [111], lack of motivation [39], [56], [57], [66], [77], [87] or damage in previous relationships [79]. Attendance for the duration of the session could also be challenging [29], [59], [62]. Coercion to attend was seen by some to have a negative impact, resulting in disruption, focus on authority, or resistance to participating and being involved in the group [27], [38], [59]. Arnason [29] also suggested that some may not feel that they need therapy or may hold ambivalence towards attending as they fear missing visitors, clinical appointments or wish to return home. Patients may attend in order to ‘play the system’ to obtain early discharge [87] and it may be that the idea of making music itself may be more motivating for patients than that of psychological change [76], [108]. Cullen [39], noted resistance may take many forms including resistance to choice, personal expression, focus on the here and now, criticism, breaking the mood of the group and intellectualization.

The papers described the therapist as highly active in identifying, informing and establishing relationships with patients both prior to, during and between sessions. Interest and willingness to work with music, level of risk and ability to function in a group were common indication criteria. Acute psychosis was seen as a contraindication by some therapists, whilst others utilized individual sessions or specific targeted groups to engage and work with this patient group. Consistency in therapeutic boundaries of environment, time, place, session structure and behaviour were seen to be of importance but could be difficult to ensure due to the availability of space and fluctuating atmosphere on the ward. When patients were unable to access the group (for example due to ward confinement), therapists would visit the patient, hold sessions on the ward, or provide taped music (either of the group session [62], or for relaxation [29] to maintain consistency of contact. Due to rapid discharge, some offered outpatient work (N = 22), or home visits where this was not possible eg. [78]. Therapists also noted the importance of preparing the groups for change or breaks.

Music therapists described greater participation and direction of the sessions. The level at which patients could influence the group process was determined by therapist approach and level of functioning of patients. Overall, papers described an approach led by patients, but structured by the therapist at the beginning and end of sessions. Opening events were used to orientate new members and closing events used for reflection. Due to the high turnover of patients, 14 papers viewed sessions as standalone sessions [29], [40], [59], [65], [79], [95], [96], [99], [100]-[103], [106], [107], [117], often influenced by the work of Yalom [121]. A range of music interventions was used. Most emphasised active musical participation, predominantly through structured improvisation and singing/playing pre-composed music. Receptive methods were used either in groups where active music making was deemed too challenging, or for higher functioning patients using a modified form of the Bonny Method of Guided Imagery in Music [31], [52], [72], [73]. Across all forms of music making, musical components of importance were described as having a clear structure, predictability, and tonal and harmonic simplicity. Musical boundaries and ground rules were employed to address behaviour within groups. Verbal reflection was described in all papers and was used to clarify and encourage communication. Discussions focused upon concrete events within the here and now with minimal interpretation.

#### Stratification by year, duration of stay and country

Year of publication and length of in-patient stay were inspected to explore whether changes to psychiatric services could have influenced the derived themes. Thirty-eight papers published between 1973 and 2011 reported lengths of stay of less than 3 months. Sixteen papers described durations over 3 months. These were published from 1995 onwards and originated from Belgium (N = 2), Denmark (N = 2), Germany (N = 3), Israel (N = 1), Norway (N = 4), and USA (N = 3). These papers all considered acute cases, but the exact length of the acute phase of treatment was not specified.

All themes covered a range of publication years and countries. Analysis of themes by publication year suggested that symptom specific aims (Aim A6) and patient turnover (Theme S4) might be more recent concerns, although it should be noted that short lengths of stay (Theme S5) covered papers published between 1975-present and there were no papers representing the years 1977–1985. Papers from Germany did not mention patient turnover as a theme of work and a single case study from Israel [94] did not mention any setting specific features of work although this may be due to the paper’s focus upon the process of therapy in relation to grief and mourning.

### Outcome Studies

Of the 35 research papers identified in this review, 10 evaluated clinical outcomes (Table 3). Of these, 8 used a randomised controlled trial design [37], [74], [84], [100], [102], [103], [116], [118], although only one utilised a reliable randomisation method [116]. Various outcomes were assessed, which were mostly social/interpersonal, mood and symptom domains.

**Table 3:** Summary of clinical outcome studies in acute adult psychiatric settings.

Paper Country	Design and Data Collection	Total number of Participants N (male), diagnosis	NEx	NCt	Mean Age (yrs)	Outcomes	Measures	Summary of intervention: Experimental (E)	Summary of intervention: Control (C)	N sessions (S), frequencyduration	Drop-outs	Bias risk QS%
**Cassity, 1976^37^ USA**	Controlled study Pre-MT, Post-MT (2 wks)	12 (0)	6	6	E: 25 C :27	Group cohesion Peer acceptance IP relations	SQ	Guitar tuition with performance plus 6 hours daily community treatment program	6 hours daily community treatment program	S = 10 Daily over 2wks	2	M 41
		8 Schizophrenia	4	4								
		1 Hyperchondrial neurosis	1	0								
		1 Depressive neurosis	1	0								
		1 Passive Dependent	0	1								
		1 Hysterical neurosis	0	1								
**Moe et al., 2000^73^ NL**	Pre- Post Pre-MT, Post-MT (6 mths)	9(7)	9		29	Global functioning	GAF	Modified Guided Imagery in Music	N/A	S = 23-32 1pw over 6 months.	0	M 59
		5 Schizotypal	5									
		3 Schizophrenia	3									
		1 Schizoaffective	1									
**Morgan et al., 2011^74^ Australia**	RCT Pre-MT, Post-MT (2wks), f/u (1 mth)	60* (49 (23) Completed) *Analysis only on completed			E: 35 C: 37	Anxiety, depression, stress Patient ward behaviour Depression Psychiatric symptoms	DASS-21 NOSIE-30 Calgary BPRS	Individual music therapy using improvisation or songwriting.	Sitting with therapist listening to a pre-recorded CD playing relaxing nature sounds.	S = 4 2pw over 2 weeks.	11 E:5 C:6	L 85
		25 Schizophrenia	11	14								
		12 Schizoaffective	6	6								
		12 Bipolar	8	4								
**Odell-Miller et al., 2006^84^** **UK**	RCT Pre-MT, Mid (3 mths), Post-MT (6mths) PQRST: Monthly.	45 (10) Individual MT = 2 Group MT = 1	nr	nr	37	Anxiety and depression Issues of importance to patient Clinical Outcomes Life skills	HADS PQRST CORE LSP	Arts therapies- Individual AT Group AT Individual DMT Individual MT* Group MT* Plus standard psychiatric support. *MT: improvisation	Standard psychiatric support.	Frequency NR 6 months	20 E:14 C:6	M 59
		9 Schizophrenia	nr	nr								
		6 Bipolar	nr	nr								
		3 Depression	nr	nr								
		3 Residual depression	nr	nr								
		2 Schizoaffective	nr	nr								
		1 Dementia	nr	nr								
		1 Eating Disorder	nr	nr								
**Silverman & Marcionetti, 2004^96^ USA**	Pre- Post Pre-MT, Post-MT (single session)	189 Gender: nr			nr	Self-reported Mood; Psychiatric symptoms; Feelings re: hospital; Self-esteem; Self-expression; Knowledge of coping skills; Managing anger; Appraisal of MT	Researcher designed 10pt VAS	5 single interventions: 1. Group drumming 2. Music games 3. Lyric analysis 4. Songwriting 5. Music listening	N/A	S = 1 Single session 2pw over 3 weeks. Each offered 8 times.	0	M 37
		Group drumming	48									
		Music Games	37									
		Lyric Analysis	34									
		Songwriting	35									
		Music Listening	35									
		Schizophrenia	nr									
		Schizoaffective	nr									
		Bipolar	nr									
		Major Depressive Disorder	nr									
		Psychosis	nr									
**Silverman, 2009a^100^ USA**	RCT Post-MT (single session)	105 Gender: nr			E: 37 C: 41	Social Functioning Patient appraisal Satisfaction with life Psycho- educational knowledge Therapist and patient verbalising in group	Researcher designed scales: 1 = worse 7 = better Helpful Enjoyment Comfort SWLS KIRI Observer rated	Opening song; Lyric analysis focusing on relapse prevention and management of mental illness	Scripted verbal psychoeducation with opening activity	S = 1 Single session 2pw over 5 months. 28 of 32 sessions attended. E = 15 sessions C = 13 sessions	App: 1 SWLS 1	L 70
		Bipolar	nr									
		Major Depressive Disorder	nr									
		Substance abuse	nr									
		Schizoaffective	nr									
		Schizophrenia	nr									
**Silverman, 2011a^102^ USA**	2 x RCTs Study 1: Pre-MT, 1 month f/u Study 2: Pre-MT, Post-MT (single session)	Study 1: 30 Study 2: 29 Gender: nr			nr	Knowledge of coping skills	PCI	Songwriting, lyric analysis and music games to address psychoeducational objectives such as coping skills, relapse prevention, leisure skills, mental health knowledge.	Psychoeducation objectives such as coping skills, relapse prevention, leisure skills, mental health knowledge without music.	Study 1: S = 3 30mins, 3pw over 4 weeks. Study 2: S = 1 45mins single session.	Study 1:21 E:11 C:10 Study 2: 0	M 63
		Bipolar	nr									
		Major depressive disorder	nr									
		Schizoaffective	nr									
		Substance abuse	nr									
		Schizophrenia	nr									
**Silverman, 2011b^103^ USA**	RCT Post-MT (single session)	89 (32) Mixed diagnoses: nr			E: 37 C: 40	Coping skills Enjoyment Therapist and Patient Working alliance	COPE Researcher designed scale 1 = Low 7 = High. HAQ-II	Opening song to state name and how feeling; Songwriting concerning coping skills using 12-bar blues.	Non-music psychoeducation group focused on coping skills.	S = 1 Single session 1pw over 4 months.	HAQ: 19	M 63
**Talwar et al., 2006^116^ UK**	RCT Pre-MT, Post-MT (3 mths)	81 (60) Schizophrenia	33	48	E:35 C:39	Positive and negative symptoms Global Functioning Patient satisfaction	PANSS GAF CSQ	Individual music therapy using improvisation and talking to guide, interpret or enhance musical experience plus routine standard care.	Routine standard care including nursing care and access to occupational, social and other inpatient activities.	S = 12 1pw over 12 weeks.	12 E:5 C:7	L 89
**Ulrich et al., 2007^118^ Germany**	RCT Pre-MT, post-MT	37 (20)	16	11	E:36 C:40	Negative symptoms IP contact: Nurse & Patient rated Quality of life	SANS GT subscales 1, 5 and 6 SPG	Structured group sessions using mainly active music making on rhythm instruments; structured improvisation, playing/singing pre-composed music, verbal reflection plus standard treatment.	Standard treatment.	S = 7-8 1-2 pw over 8 months. Average n sessions received = 7.5 (sd 3.5)	SANS: E:5 C:5 GTN: E:0 C:3 GT-P: E:4 C:3 SPG: E:4 C:2	L 82
		27 Schizophrenia	16	11								
		4 Schizoaffective	3	1								
		1 Schizotypal	0	1								
		3 Drug induced psychosis	2	1								
		2 Depression with psychosis	0	2								

BPRS- Brief Psychiatric Rating Scale, Calgary- Calgary Interview Guide for Depression, COPE- Brief COPE Inventory, CORE- Clinical Outcomes in Routine Evaluation, CSQ- Client Satisfaction Questionnaire, DASS-21- Depression, Anxiety and Stress Scale, GAF- Global Assessment of Functioning Scale, GT- Gießentest , HADS- Hospital Anxiety and Depression Scale, HAQ-II- Helping Alliance Questionnaire, KIRI- Knowledge of Illness and Resources Inventory, LSP- Life Skills Profile, NOSIE-30- Nurses’ Observation Scale for Inpatient Evaluation, PANSS- Positive and Negative Symptoms Scale, PCI- Proactive Coping Inventory, PQRST- Personal Questionnaire Rapid Scaling Technique, SANS- Scale for the Assessment of Negative Symptoms, SPG- Scales for Mental Health, SWLS- Satisfaction with Life Scale, SQ- Sociometric Questionnaire, VAS- Visual Analogue Scale

AT- Art Therapy, DMT- Dance Movement Therapy, MT, Music Therapy, nr- Not reported, N/A- Not applicable, pw- per week, IP relationship- Interpersonal Relationships

#### Risk of bias within studies

Six studies were evaluated as medium quality (37%–62.9%) [37], [73], [84], [96], [102], [103] and four as high (70.4%–88.9%) [74], [100], [116], [118] (Table 4). Studies were strong in reporting, but had significant shortcomings in four areas: Information regarding adverse events was reported in only one study [73]; six did not outline explicit exclusion criteria [37], [84], [96], [100], [102], [103]; four did not provide a description of principle confounders [37], [84], [96], [103] and three did not report characteristics of patients lost to follow-up [37], [96], [100]. External validity was difficult to assess as only two studies provided adequate information regarding the source population, selection of patients and the proportion of those invited who agreed [74], [116]. Internal validity was limited by a lack of blinding of subjects, outcome assessors, and concealment of randomisation with only one study adequately addressing these [74].

**Table 4:** Risk of bias of included clinical outcome studies.

Study:		Cassity 1976 ^37^	Odell-Miller et al 2006^84^	Silverman & Marcionetti 2004^96^	Silverman 2009a^100^	Silverman 2011a^102^	Silverman 2011b^103^	Talwar et al. 2006^116^	Ulrich et al 2007^118^	Moe et al 2000^73^	Morgan, et al 2011^74^
Experimental Study Design		Controlled study	RCT	Pre-Post	Controlled study	Pilot RCT	2 Pilot RCTs	RCT	RCT	Pre-Post	RCT
Score/27 (%) (Downs & Black, 1998)		11 (40.7)	16 (59.3%)	10 (37%)	19 (70.4%)	17 (62.9%)	17 (62.9%)	24 (88.9%)	22 (81.5%)	16 (59.3%)	23 (85.2%)
Reporting	Hypothesis/aim/objective	Yes	No	Yes	Yes	Yes	Yes	Yes	Yes	Yes	Yes
Clear description of-	Main outcomes to be measured	Yes	Yes	Yes	Yes	Yes	Yes	Yes	Yes	Yes	Yes
	Patient characteristics	No explicit exclusion criteria	No explicit exclusion criteria	No explicit exclusion criteria	No explicit exclusion criteria	No explicit exclusion criteria	No explicit exclusion criteria	Yes	Yes	Yes	Yes
	Intervention	Yes	No	Yes	Yes	Yes	Yes	Yes	Yes	Yes	Yes
	Distribution of principal confounders	No	No	No	Yes	Yes	No	Yes	Yes	Yes	Yes
	Main findings	No data for subject rank	Yes	Yes	Yes	Yes	Yes	Yes	Yes	Yes	Yes
	Estimates of random variability in data for main outcomes	Not reported	Yes	Yes	Yes	Yes	Yes	Yes	Yes	Yes	Yes
	All adverse events	No	No	No	No	No	No	No	No	Yes	No
	Characteristics of patients lost to followup	Not reported	Yes	Not reported	Not reported	Yes	Yes	Yes	Yes	Yes	Yes
	Actual probability values reported	Yes	Yes	No	No	Yes	Yes	Yes	Yes	Not reported	Yes
External Validity	Subjects approached representative of entire population	Unable to determine	Yes	Unable to determine	Yes	Unable to determine	Yes	Yes	Unable to determine	Unable to determine	Yes
	Participants representative of entire population	Unable to determine	Unable to determine	Unable to determine	Unable to determine	Unable to determine	Unable to determine	Yes	Unable to determine	Unable to determine	Yes
	Staff and facilities representative of treatment usually received	Yes	Yes	Yes	Yes	No	Yes	Yes	Yes	Yes	Yes
Internal validity	Subjects blinded to intervention	No	No	No	No	No	No	No	No	No	Yes
(bias)	Measurers of main outcomes blinded	No	Yes	No	No	No	No	Yes	Yes	No	Yes
	Unplanned analyses reported	No unplanned analyses	Yes	No unplanned analyses	Yes	No unplanned analyses	Yes	No unplanned analyses	No unplanned analyses	No unplanned analyses	No unplanned analyses
	Adjustment of different lengths of follow-up	Same time period for follow up	Same time period for follow up	Same time period for follow up	Same time period for follow up	Same time period for follow up	Same time period for follow up	Yes	Same time period for follow up	Same time period for follow up	Same time period for follow up
	Appropriate statistical tests to assess main outcomes	Yes	Yes	Unable to determine	Yes	Yes	Yes	Yes	Yes	Yes	Yes
	Reliable compliance with intervention	Unable to determine	Yes	Yes	Yes	Unable to determine	Yes	Yes	Yes	Yes	Unable to determine
	Accurate outcome measures (valid and reliable)	Yes	Yes	Yes	Yes	Yes	Main outcome valid and reliable. Additional measures not outlined in method.	Yes	Yes	Yes	Yes
Internal validity (confounds)	Recruitment of intervention and control from same population	Yes	Yes	No control group	Yes	Yes	Yes	Yes	Yes	No control group	Yes
	Recruitment over same period of time for control and intervention	Yes	Yes	No control group	Yes	Yes	Yes	Yes	Yes	No control group	No- quasi random by month
	Randomisation to groups	Unable to determine	No- alternate allocation	No	Yes	No- quasi random by intervention	Study 1- unable to determine; Study 2- randomised by session	Yes- block randomised stratified for site, derived from computer program	Yes- randomised to intervention or control by throw of dice	Not randomised	No- quasi random: 1 month intervention then 1 month control
	Concealment of randomisation	Unable to determine	No	Not randomised	No	No	No	Concealed from staff, not patients	Concealed from staff, not patients	Not randomised	Yes
	Adjustment for confounding	No	No	No	Yes	No statistically significant differences between groups regarding number of times in hospital or age.	Yes	Yes	Yes	No	No significant differences between groups
	Loss of patients to follow up taken into account	No	Yes	No	Yes	Yes	Yes	Yes	Yes	Yes	Yes

#### Risk of bias across studies

In terms of missing studies, one protocol was identified which did not have ensuing published data [122]. It was unclear whether this study involved acute in-patients [122]. Only one study protocol was available to examine selective reporting bias [83], of which all outcomes were reported in the final publication [84]. One study [103] reported outcomes not explicitly outlined in the method whilst Cassity [37] did not provide tabulated data for measures of peer acceptance and interpersonal relationships.

#### Clinical Outcomes

Comparisons of clinical outcomes are shown in Table 5. Direction and size of pre- post- change in the intervention group, post intervention differences between groups and statistical significance were examined to compare the strength of evidence between studies. Reductions in positive and negative symptoms [74], [116], [118], psychiatric symptoms [74] and increased interpersonal functioning [118] were significantly more favourable in patients receiving music therapy compared to controls, although the size of the effects were small. All used active music-making methods with a degree of structure and delivered between 4–12 sessions over 2 weeks to 3 months. These studies were of a higher methodological quality than most of the studies in this review. However, studies were limited by lack of blinding of interviewers, small sample sizes (N = 12–81), and few used an active control.

**Table 5:** Comparison of outcomes (standardised mean difference) across studies and vote count.

Outcome	Study	Measure	Session content	Technique	N sess	SMD Pr-Po	SMD E vs C	E	G	S	Bias risk
**Social and interpersonal outcomes**											
Interpersonal contact- patient rated	Ulrich et al 2007	GT Patient	Active	Improvisation, pre-composed	7.5	0.41	0.64*	+	+	+	L
Interpersonal contact- nurse rated	Ulrich et al 2007	GT Nurse	Active	Improvisation, pre-composed	7.5	0.18	0.25	+	+		L
Social Functioning	Silverman 2009a	RD Scale	Receptive	Lyric analysis	1	na	0.09	+	+		L
Interaction	Morgan et al 2011	NOSIE Interaction	Active	Improvisation, songwriting	4	0.5	−2.30	+	-		L
Working alliance- therapist	Silverman 2011b	HAQ-II Therapist	Active	Songwriting	1	na	1.09*	+	+	+	M
Working alliance- patient	Silverman 2011b	HAQ-II Patient	Active	Songwriting	1	na	0.31	+	+		M
**Dichotomous Social and interpersonal**											
Group cohesion	Cassity 1976	SQ	Active	Didactic	10	1.12	0.98	+	+		M
Peer Acceptance	Cassity 1976	SQ	Active	Didactic	10	0.76	0.61	+	+		M
Interpersonal relations	Cassity 1976	SQ	Active	Didactic	10	nr	nr				M
**Global functioning**											
	Talwar et al 2006	GAF	Active	Improvisation, verbal reflection	12	0.43	0.13	+	+		L
	Moe et al 2000	GAF	Receptive	Modified GIM	28	1.22*	na	+		+	M
**Increased quality of life**											
	Ulrich et al 2007	SPG	Active	Improvisation, pre-composed	7.5	0.24	0.05	+	+		L
**Global distress**											
	Odell-Miller 2006	CORE	Active	Improvisation	nr	0.09	0.02	-	-		M
**General psychiatric symptoms**											
	Morgan et al 2011	BPRS Total	Active	Improvisation, songwriting	4	−1.07	−0.16*	+	+	+	L
	Talwar et al 2006	PANSS General	Active	Improvisation, verbal reflection	12	−0.71	−0.32	+	+		L
**Negative symptoms**											
	Morgan et al 2011	BPRS -ve symptoms	Active	Improvisation, songwriting	4	−1.43	−0.03*	+	+	+	L
	Ulrich et al 2007	SANS Total	Active	Improvisation, pre-composed	7.5	−0.53	−0.42*	+	+	+	L
	Talwar et al 2006	PANSS -ve symptoms	Active	Improvisation, verbal reflection	12	−0.56	−0.30	+	+		L
**Positive symptoms**											
	Morgan et al 2011	BPRS+ve symptoms	Active	Improvisation, songwriting	4	−1.08	−0.24*	+	+	+	L
	Morgan et al 2011	NOSIE Psychosis	Active	Improvisation, songwriting	4	−0.67	−0.10	+	+		L
	Talwar et al 2006	PANSS+ve symptoms	Active	Improvisation, verbal reflection	12	−0.67	−0.28	+	+		L
**Positive and negative symptoms**											
	Talwar et al 2006	PANSS Total	Active	Improvisation, verbal reflection	12	−0.66	−0.26*	+	+	+	L
**Depression**											
	Morgan et al 2011	BPRS Depression	Active	Improvisation, songwriting	4	−1.06	−0.05*	+	+	+	L
	Morgan et al 2011	Calgary	Active	Improvisation, songwriting	4	−0.63	−0.04	+	+		L
	Morgan et al 2011	DASS-21	Active	Improvisation, songwriting	4	−0.51	0.02	+	−		L
**Anxiety and depression**											
	Odell-Miller 2006	HADS	Active	Improvisation	nr	−0.12	0.15	+	−		M
**Dissociation**											
	Morgan et al 2011	BPRS Dissociation	Active	Improvisation, songwriting	4	−0.73	−0.12*	+	+	+	L
**Mania**											
	Morgan et al 2011	BPRS Mania	Active	Improvisation, songwriting	4	−1.2	−0.13*	+	+	+	L
**Anxiety**											
	Morgan et al 2011	DASS-21	Active	Improvisation, songwriting	4	−0.83	−0.10	+	+		L
**Stress**											
	Morgan et al 2011	DASS-21	Active	Improvisation, songwriting	4	−0.71	−0.22	+	+		L
**Irritability**											
	Morgan et al 2011	NOSIE Irritability	Active	Improvisation, songwriting	4	−0.5	0.13	+	−		L
**Ward behaviour**											
	Morgan et al 2011	NOSIE Total	Active	Improvisation, songwriting	4	0.46	−0.08	+	+		L
**Life skills**											
	Odell-Miller 2006	LSP	Active	Improvisation	nr	−0.3	−0.63	−	−		M
**Psychoeducational knowledge**											
	Silverman 2009a	KIRI	Receptive	Lyric analysis	1	na	0.08		+		L
**Coping skills**											
	Silverman 2011a	Study 1: PCI	Mixed	Songwriting, lyric analysis, music games	3	na	1.52		+		M
	Silverman 2011a	Study 2: PCI	Mixed	Songwriting, lyric analysis, music games	1	na	0.12		+		M
	Silverman 2011b	COPE	Active	Songwriting	1	na	0.03		+		M
**Appraisal and satisfaction**											
Increased satisfaction with services	Talwar et al 2006	CSQ	Active	Improvisation, verbal reflection	12	0.34	0.33	+	+		L
Increased enjoyment	Silverman 2011b	RD Scale	Active	Songwriting	1	na	0.09		+		M
Increased enjoyment	Silverman 2009a	RD Scale	Receptive	Lyric analysis	1	na	0.15		+		L
Increased helpfulness	Silverman 2009a	RD Scale	Receptive	Lyric analysis	1	na	0.09		+		L
Increased satisfaction with life	Silverman 2009a	SWLS	Receptive	Lyric analysis	1	na	0.24		+		L
Increased comfort	Silverman 2009a	RD Scale	Receptive	Lyric analysis	1	na	−0.08		−		L
Vote count coding key											

Experimental group pre-post SMD E	SMD Group difference post intervention G	Outcome is statistically significant S	Interpretation
+	+	+	Significantly favours intervention
+	+		Trend towards intervention
+	−		Improvement in experimental group, trend towards control
−	−		Deterioration in experimental group, trend towards control
−	−	−	Significantly favours control

+Direction of experimental pre-post SMD indicates improvement/group difference SMD favours intervention/significantly favours intervention.

− Direction of experimental pre-post SMD indicates deterioration/group difference SMD favours control/significantly favours control.

* Statistically significant (*p*<.05).

N sess- Number of sessions received, SMD- Standardised mean difference, Count- Vote count, nr- not reported, na- not applicable.

BPRS- Brief Psychiatric Rating Scale, Calgary- Calgary Interview Guide for Depression, COPE- Brief COPE Inventory, CORE- Clinical Outcomes in Routine Evaluation, CSQ- Client Satisfaction Questionnaire, DASS-21- Depression, Anxiety and Stress Scale, GAF- Global Assessment of Functioning Scale, HADS- Hospital Anxiety and Depression Scale, HAQ-II- Helping Alliance Questionnaire, KIRI- Knowledge of Illness and Resources Inventory, LSP- Life Skills Profile, NOSIE- Nurses' Observation Scale for Inpatient Evaluation, PANSS- Positive and Negative Symptoms Scale, PCI- Proactive Coping Inventory, RD- Researcher designed, SANS- Scale for the Assessment of Negative Symptoms, SMD- Standardised mean difference, SPG- Scales for Mental Health, SQ- Social Questionnaire, SWLS- Satisfaction with Life Scale.

Vote count coding key: Positive outcomes suggesting a trend towards the intervention but not significant when compared to controls included patient behaviour on the ward (NOSIE-30), patient experienced anxiety and stress (DASS-21) [74], global functioning (GAF), satisfaction with music therapy (CSQ) [116] and quality of life (SPG) [118]. Three randomised controlled trials by Silverman [99], [102], [103] examined the effect of psychoeducational music therapy interventions upon psychoeducational knowledge**,** coping skills, satisfaction with life and appraisal of music therapy after a single session. Sessions were based on a psychoeducational framework with the aim to educate patients with knowledge and skills to manage their mental illness. Interventions included lyric analysis, songwriting and music games, with themes of relapse prevention, management of mental illness, active coping strategies for common problems faced, leisure skills and improving mental health knowledge. The active control followed the same psychoeducational script but did not employ music activities. Patients demonstrated greater psychoeducational knowledge in the music therapy group compared to the control in all three studies but these were not statistically significant. Effects may have been limited by assessment of a single session and use of an active control.

Outcomes for depression were mixed. Morgan et al. [74] found treatment group BPRS scores significantly decreased compared to the control. However, reduction was not significant compared to the control when assessed on the Calgary Interview Guide for Depression whereas scores on the DASS-21 suggested a trend towards the control. Other outcomes with a trend towards the control group were irritability and interaction subscales of the NOSIE-30, [74] and ratings of comfort after a single session of psychoeducational lyric analysis [99].

Odell-Miller et al’s study [84] on the effectiveness of arts therapies (music, dance movement and art therapy) compared 10 patients receiving an arts therapy intervention, to 15 patients receiving treatment as usual at three time points. Patients in the treatment group reduced in anxiety and depression but this was not significant and the group difference favoured the control. Individual global distress reduced in both treatment and control, but increased in the final assessment for the treatment group. Life skills increased for the control, but decreased in the treatment group. Despite its rigorous design, the authors noted the problems inherent in assessing a range of interventions, diagnoses, and small number of participants.

#### Subjective outcomes

Five papers sought patient evaluations of music therapy. Reker [90], Heaney [60] and Dye [44] used questionnaire-based surveys. Silverman [101] combined a questionnaire and interview to ascertain patient perceptions of different interventions, whilst Ansdell & Meehan [28] conducted in-depth idiographic interviews.

Reker [90] designed a 25 item questionnaire for patients to rate their experience of active music therapy utilising structured music making, and 30 patients completed the questionnaire. Patients rated music therapy positively, particularly in terms of enjoyment, safety relaxation and improvement in mood. Patients noted that it was anxiety provoking to play, although only 5 respondents partly felt that the music made them uneasy or frightened. Patients found it difficult to speak about the music although all rated that it was important to speak about the music after playing. Dye [44] found patients rated both a singing and listening group highly, with slightly higher ratings for the singing group. Out of 39 responses, all but one were able to suggest a song that was meaningful for them during the session. Dye notes the consensus between individuals for favoured songs in the group, although personal reasons given as to why these songs were favoured varied between individuals. In his comparison of music therapy to other group therapies, Heaney [60] examined ratings from 27 patients. He found music therapy consistently gained the most positive appraisals, and was significantly more pleasurable than other groups, whilst there were no significant differences in importance and success ratings. Heaney found a relationship approaching significance for age, but no significant relationships between overall ratings and length of admission or previous hospitalisation.

When assessing patient perceptions of 5 psychoeducational interventions (individual game, team game, singalong session, lyric analysis, songwriting), Silverman [101] found patients rated the team game as most enjoyable and individual games least. However, the individual game had highest helpfulness ratings and lyric analysis the lowest. Whilst patients could recall events in the group, they were not always able to state what the purpose of the group had been. All stated they would attend another session.

Ansdell and Meehan’s study [28] revealed in greater depth the experiences of patients who had significantly engaged in music therapy for a minimum of 10 individual sessions. The study met all but two of the qualitative framework criteria (attention to (12c) and explanation of (14d) negative cases, outliers or exceptions) [24]. Nine themes were defined: 1. Benefit is broader than symptomatic change; 2. Music therapy often involves reconnecting with a previous relationship to music; 3. Music therapy elicits and works with patients’ “music-health-illness” narrative; 4. Qualities of ‘musical’ and ‘therapeutic’ are often experienced as a unity; 5. Aspects of musical process in music therapy are experienced as distinctive; 6. The therapist is experienced as an equal ‘musical companion’; 7. Music therapy is experienced as distinctive in relation to other therapies; 8. Overall benefits are characterised as compensatory or alleviatory in relation to illness experiences; 9. A key benefit of music therapy is its ability to mobilise “music’s hope”. They suggest that the “music-health-illness” narrative forms 3 parts whereby patients have a previous positive relationship and history with music, which is lost when becoming ill, leading to loss of music as a helping resource. The authors suggest that the accounts indicate music therapy enables this relationship to be re-established, thus providing patients with a means of seeking help from music themselves again.

## Discussion

This review has identified a wide variety of ways in which music therapists work within acute adult psychiatric settings. Therapists respond to the challenges of the setting and system, as well as the diverse and individual needs of the patients. Initial engagement of patients with therapy is a core aim and emphasis is placed upon immediate presenting emotional, interpersonal and behavioural issues. Whilst papers describing clinical practice have some shared features which may be of importance for work in these settings, it is clear that as yet, no clearly defined model exists to accommodate the challenges of providing music therapy in acute adult mental health care.

### Patient and Setting Challenges

The short period of in-patient stay has been a challenge for music therapists working to models that assume a longer period of work. Combined with the severity and range of symptoms, attendance and engagement were of particular concern. Adaptations to address this include increased session frequency, viewing sessions as standalone, targeted groups for particular function levels or needs and service diversification to incorporate the wider hospital, outpatients and community.

There is mixed evidence concerning attendance and engagement of patients in music therapy. The outcome studies suggest that adherence to music therapy is high, although this is contrasted with the difficulties in managing rapid patient turnover and fostering initial engagement. Despite the acknowledged difficulties in fostering group processes due to high turnover, few papers have fully examined the impact of this upon engagement in therapy. The early stages of group development described by Hara [59], Jensen [64] and Hannibal [57] fit with those described within acute verbal psychotherapy groups [123] and the early stages of wider music therapy mental health groups [124]. Further research into the impact of group processes and music therapy techniques upon engagement in music therapy is therefore required.

### Clinical Aims

Aims focus upon fostering therapeutic engagement with patients, building interpersonal relationships and immediate effects such as reduction in arousal or relaxation, which were suggested to be of immediate benefit both to individuals, and the ward environment as a whole. Patients within acute settings were noted to be in crises, and interventions therefore focused upon management of symptoms and interpersonal relationships in the ‘here and now’ rather than long term insight or understanding.

The lack of clear indication criteria and diagnostic focus is problematic for therapists working in acute settings as evidenced by the difficulties in communicating the value and purpose of music therapy to the multi-disciplinary team. This has been a wider issue for some time in music therapy mental health work [125]-[127]. The findings suggest that the patient’s interest and willingness to work with music, level of risk and ability to function in a group were core criteria. Music therapists may need to articulate their aims and criteria for referral with an emphasis upon immediate and short term benefits, along with ways in which patients might access and benefit from medium and longer term services.

Despite the heterogenous delivery of music therapy, different processes may be apparent for distinct diagnoses. Within this review, de Backer [10], Jensen [64] and Solli & Rolvsjord [110] suggest distinct ways of working with acute psychosis, which was seen by some other authors as a contraindication for wider mixed groups. In the wider mental health literature, distinct patterns and processes within musical co-improvisation have been identified in depression and schizophrenia but this does not appear to have been explored any further within acute clinical practice [128]. In contrast, those utilizing resource-oriented principles, use methods to support and strengthen patient engagement in music and argue against a purely diagnostic focus.

### Diversity of Practice

Approaches to music therapy were diverse, influenced by training and predominating models of their country. Previous reviews have also noted the diversity of practice and approaches in mental health [4], [6], [7], [9]. Music therapy approaches often conflicted with the changing institutional models and structure. For example, within the UK, two early music therapists, Fenwick and Priestley [48], [86], describe services that offered music therapy to the whole hospital as an institution, such as open ensembles for staff, in- and outpatients. Later work by Grandison [54] and Odell-Miller [12], [82] details the challenges faced as hospitals moved from therapeutic communities, conducive to group work models of music therapy on acute admission wards, to shorter term individually delivered medical models. A similar situation is seen in the work of Silverman in the USA, whereby approaches were adapted to fit with the institution’s psycho-educational and short term model.

Later papers in this review suggest that patient and setting specific models are beginning to evolve. Papers from the 1980s onwards are influenced by Yalom’s application of group psychotherapy to in-patient settings [121], [129], whilst therapists working within psychoanalytic and psychodynamic approaches have adapted their models to focus more upon the immediate interpersonal processes (influenced by the work of Daniel Stern [130], [131]) than upon interpretation of transference dynamics. In Norway, the concept of resource orientation in mental health care is also developing [13], [76], [107]-[110].

### Outcome Studies

Few studies have rigorously evaluated the effectiveness of music therapy specifically for acute psychiatric in-patients. The studies in this review provide some evidence suggesting that active music therapy can be effective in reducing psychiatric, positive and negative symptoms and improving interpersonal interaction although the length of time evaluated in these studies is generally much greater than typical lengths of in-patient stay. Studies of shorter durations suggest minor improvements, but these are not sustained at follow-up. Morgan et al. [74] note that the short time frame of therapy (2 weeks) might explain the lack of significant findings in their study. Similarly, studies of the immediate effects of psychoeducational music therapy [99], [102], [103] suggest minor improvements in a range of areas including coping skills, but these were not significant after 3 months. These findings are in line with the suggested dose-effect response [4] yet it remains unclear as to what role the immediate effects of primarily active music making and frequency of sessions may have upon processes and outcomes for this patient group.

Structured active music production, such as structured improvisation or active playing of pre-composed music plays a dominant role in music therapy for this patient group and was supported in the findings from outcome studies. Whilst active techniques were dominant across all countries, 11 papers observed that playing music actively could provoke high anxiety in patients. Therapists attempted to alleviate this by providing information and reassurance prior to the group, structured activities, and music familiar to patients. A recent study of music therapy techniques as predictors of change in individual work with adults with severe mental health problems and low motivation [132] found that use of music reproduction techniques, such as playing precomposed music or tuition of basic musical skills was associated with gains in relational competencies. The authors suggested that the pre-formed musical structure in music reproduction (ie. actively playing pre-composed music) can support patients who find it difficult to express or create their own music. Similarly, in her study of art and dance movement therapy, Dokter [133] found that young adults in a longer term therapeutic community setting valued active participation, but therapists had to carefully manage structure, discussions and arts activities to counter initial anxiety, meet individual needs and the stage of the group. It may be that use of familiar musical structures and styles assists in alleviating anxiety and builds the confidence to nonverbally further explore emotions and relationships in music [13], [108], [132].

If both engagement and clinical improvement are dependent on 20 or more sessions [4], [58] one might still question what value music therapy in acute settings may have in the treatment process. Whilst some papers cited the importance of fostering therapeutic engagement for longer term work in the community, this review identified difficulties in the linkup between in-patient and outpatient services, and the lack of continuation when outpatient work was offered. It is unclear to what extent patients are able to access further therapy after discharge and what impact this might have upon outcomes. Small benefits can be seen after a single session, but do not reach statistical significance. However, studies which incorporated patient feedback provided evidence of positive appraisals of music therapy with emphasis on improvement in mood, relationships and fostering of motivation.

### Implications for Future Research

This review has identified clinical practice spanning 40 years across a range of countries in acute in-patient settings. Despite this large body of work, very little research exists to qualify the evidence base for practice in acute settings. One possible model of music therapy may be to offer a high intensity of sessions. However, whilst evidence suggests a greater number of sessions is required to achieve clinically significant benefits, no research has yet assessed whether increasing the frequency of therapy is accepted by patients. An alternative or adjunctive model may be to focus on brief interventions lasting only a few or even a single session although this would require consideration of clinical aims and outcomes that might be possible to achieve in such a short amount of time. Future research needs to disentangle the processes of music therapy for this population in order to better define indications and the types of outcomes that may be achieved. Development of models with consistent aims, theoretical concept and delivery is required if feasibility and effectiveness of music therapy within these settings is to be tested in systematic research including randomized controlled trials. Such developments would assist in defining the role, purpose and effective clinical practice of music therapy in acute in-patient settings.

### Strengths and Limitations

To our knowledge this is the first systematic review of clinical practice of music therapy in acute adult psychiatry. The review employed a rigorous methodology, with a wide search strategy and systematic quality appraisal. The range of identified papers was large and the use of thematic synthesis ensured that the analysis was fully grounded in the data presented. Core themes within the analytic framework of clinical considerations and aims are represented internationally, indicating robustness of the synthesis, although the manner in which therapists adapted practice varied according to approach and country.

Despite the rigorous methodology, there are a number of limitations. Whilst the scope was wide to detect variations in clinical practice, the small number of research papers and inclusion of low quality research designs means that little can be concluded regarding effectiveness. Meekams & Daniels [134] note the challenges in combining quantitative and qualitative data within thematic synthesis. The majority of papers identified in this review came from secondary searches performed after searching of electronic databases. The review only identified four studies that would meet more rigorous criteria for meta-analysis of clinical outcomes, each of which employed diverse music therapy methods, and outcome measures. Within an acute in-patient setting, evaluation of music therapy as only one part of treatment is problematic given that patients are treated within the whole institution and are seen to improve rapidly to a point where they can be discharged. Given the extremely wide nature of the review, the full depth of papers, particularly within case studies is not covered. Papers from the Far East and Asia were under-represented with 3 of the unobtainable papers originating from these countries, and this review may have missed other important and potentially different ways of working.

### Conclusion

The review suggests that currently there is no agreed, well researched and evidenced, clearly defined model of music therapy that accommodates the challenges of acute adult psychiatric in-patient settings. Changes to service setup have resulted in a need to modify existing models of music therapy to focus upon immediate and short term aims. Features of music therapy which may play an important role for this context include the frequency of therapy, active structured music making with verbal discussion, consistency of contact and boundaries, an emphasis on building a therapeutic relationship and building patient resources. Further research is now needed to develop clear models and aims, which take into account the acute in-patient context and provide information on the varying processes and outcomes. Such a model would provide greater clarity on the role and purpose of music therapy for acute adult in-patients and would provide a better defined framework of practice which can be tested in clinical trials.

## Supporting Information

Information S1 Review protocol.
(DOC)

Information S2 Search sources and example of search strategy.
(DOCX)

Information S3 Data extraction form.
(DOC)

Information S4 Paper characteristics.
(DOCX)

Information S5 Coverage of themes.
(DOCX)

Information S6 Analysis of client and setting characteristics.
(DOCX)
