# Supplementary material for: A Systematic Review of Music Therapy Practice and Outcomes with Acute Adult Psychiatric In-Patients
Source: PLoS One. 2013 Aug 2;8(8):e70252. doi: 10.1371/journal.pone.0070252 (PMC3732280; doi:10.1371/journal.pone.0070252)
Supplement: Information S1 — Review protocol. (DOC) [file pone.0070252.s001.doc]

**Supporting Information 1. Review Protocol**

**Systematic Review of Music Therapy for Inpatients in Acute Psychiatric Hospitals**

**Background:**

Music therapy as an intervention in acute psychiatric hospitals:

Music therapy has been routinely offered within UK healthcare over the last 50 years. Recent clinical guidance suggests that the arts therapies are currently the only intervention to demonstrate effectiveness in reducing negative symptoms of schizophrenia (NCCMH, 2010). There is evidence to suggest that music therapy is helpful in reducing symptoms of schizophrenia in the short to medium term, with particular impact upon negative symptoms.

Music therapy is an intervention which uses different forms of music making within the context of a therapeutic relationship. Music may be actively produced, most commonly through improvisation; or receptive, for example, listening to pre-recorded music brought by the patient. The type of musical interaction, level of structure and amount of verbal discussion may vary depending upon the music therapist’s approach, client characteristics and diagnosis. Models of music therapy vary in theoretical underpinning and approaches, but all place a relationship between therapist and client at the heart of the intervention. This is in contrast to studies of music interventions, where music is used for its specific effects. Interventions can take the form of group or individual therapy and aims will vary according to the specific needs of the patient.

Current schizophrenia guidance for arts therapies interventions recommends group therapy as a first-line treatment with creative activities being unstructured and led by the patient (NCCMH,2010). Aims are broadly outlined as:

1. To enable different experiences of oneself and to develop new ways of relating to others
2. To help self-expression and organisation of experience into a satisfying aesthetic form
3. To help people accept and understand feelings arising from the creative process at a pace suited to the person

(NCCMH, 2010; p.370)

However, the extent to which arts therapists work within these guidelines and variance across settings is unclear. Two studies have found that methods of treatment for adults with mental disorders vary considerably and a there is a lack of consistent evidence to support or contraindicate a particular approach or technique (Drieschner & Pioch, 2002; Odell-Miller, 2007). However, there does seem to be strong agreement between music therapists for the use of supportive psychotherapeutic approaches in work with psychotic disorders, with less-structured techniques of free improvisation with talking and use of pre-composed songs featuring most prominently (Drieschner & Pioch, 2002; Odell-Miller, 2007).

Effectiveness of music therapy in the treatment of severe mental illness

Two systematic reviews of effectiveness of music and music therapy for schizophrenia and related disorders have been conducted to date (Gold, Dahle, Heldal & Wigram, 2006; Silverman, 2003). Gold et al. (2006), found that music therapy was superior to standard care alone for global state, general mental state, negative symptoms and social functioning. However, effects were not consistent across studies and were dependent on the number of music therapy sessions received. Studies utilising music therapy interventions were included, and studies using only music listening were excluded. The authors defined the activities of music therapy based upon Drieschner and Pioch (2001) as:

1. Active versus receptive
2. Level of structure
3. Focus of therapeutic attention

All three variables varied across the studies included in the review.

Silverman’s meta-analysis reviewed any quantitative research that evaluated ‘the influence of music upon the symptoms of psychosis’. Inclusion criteria were more open than that of Gold et al. (2006) and included any type of music intervention, including background music and music listening. Aimed functions of the music intervention (for example, to decrease auditory hallucinations or increase appropriate behaviours) were included as part of the analysis. Nineteen quantitative studies were identified, although only one study utilised music therapy as commonly practised within the UK. The meta-analysis found differences in results from patients with catatonic symptoms (defined as ‘involving participation, cooperation, and interaction’) compared to general and cognitive symptoms. No differences in effectiveness were found between music therapy techniques and passive listening. Moreover, effectiveness was significant across all aimed functions of music. No differences were found between long-term or short-term institutions leading the author to conclude that music might therefore have an ‘immediate effect on all types of patients’ (p.37). Both analyses demonstrated effectiveness across all music therapy interventions, yet it is unclear to what extent the type of intervention (eg. Active/receptive; level of structure) impacts upon effectiveness.

More recently, Odell-Miller conducted a systematic review of music therapy techniques for specific diagnoses for her PhD thesis (2007) and Gold, Solli, Krüger & Lie have performed a meta-analysis of music therapy for people with serious mental disorders including psychosis (2009). Whilst Odell-Miller provides the first comprehensive overview of techniques utilised for specific diagnoses, it is clear that to date, there is no overall consensus as to which specific music therapy interventions might be most helpful or harmful for patients with psychosis. More specifically, there is little distinction between interventions offered for adults in acute stages of illness (most often as inpatients), and those offered long-term in the community. There is evidence to suggest a dose-effect response in the treatment of severe mental disorders, with effects depending on the number of sessions received (Gold, Solli, Krüger & Lie, 2009). The authors note that a greater number of sessions is required for ‘stronger, clinically more meaningful’ effects but it is yet unclear what impact the frequency of sessions and time period will have upon effects (Gold et al., 2009; p.205).

An initial search of PUBMED, PsychINFO and the journal ‘The Arts in Psychotherapy’ using ‘music therap*’ AND ‘psychosis’ yielded 128 papers, many of which were published post-2006. The most recent systematic review of effectiveness is now 5 years old, therefore it will be important to also consider more recent study findings.

Whilst Odell-Miller (2007) provides the first comprehensive overview of techniques utilised for specific diagnoses, it is clear that to date there is little distinction between interventions offered in acute stages of illness, and those offered long-term. It is also unclear as to whether music therapy techniques have been adapted for either shorter lengths of inpatient stay or for increased frequency of therapy.

**Methods:**

**Objectives:**

To identify adaptations of music therapy for use with acute adult psychiatric inpatients.

1. What are the clinical aims and considerations for music therapy with acute adult psychiatric patients in acute hospital settings?
2. How is music therapy provided in these settings in terms of frequency, duration and methods used?
3. What are the findings from outcome studies conducted in these settings?

**Criteria for selecting studies for this review:**

**Population:**

An initial search will look at use of music therapy with adult inpatients admitted to mental health hospitals. The review will then focus upon acute adult inpatients (ages 18+). Patients with comorbid disorders will be included and comorbidities noted. Mixed diagnostic groups will be included.

**Intervention:**

Interventions of any length, using music therapy as the main component of treatment, that utilise a mixture of active and receptive techniques will be included. Studies using passive listening, without additional therapeutic intervention, such as background music will be excluded. Studies involving concurrent interventions will be included and the concurrent interventions noted. Interventions where music therapy is delivered by a professional other than a music therapist will be included in the review if the intervention is assessed to meet the criteria as defined above.

**Outcomes:**

Any outcomes of clinical and therapeutic importance including diagnosis specific symptoms, general symptoms, motivation, attendance, musical engagement, musical preference, social and behavioural changes resulting from a particular music therapy intervention will be extracted and subject to narrative synthesis. Outcomes will be classified as positive change, no change, adverse events, or not reported. Any outcomes measured by rating scales will be reported as part of the review and linked to frequency and techniques when possible including diagnosis specific symptoms, general symptoms and mood.

**Study Design:**

As this is primarily a qualitative review to assess adaptation of techniques for increased frequency of therapy, all study designs will be included. Where prospective studies have been undertaken, study design and quality will be evaluated as part of the extraction process and included in the synthesis. No publication year or language restrictions will be applied.

**Search Strategy:**

1. Database search using search terms:

[* musi* or musi* or * sound* or sound* or * acou* or acou* or

gim in title, abstract, index terms of REFERENCE] or [music* in

interventions of STUDY] and [psychiatr* or mental*]

Followed by a hand search of studies within acute hospitals.

NB: ‘gim’ is included to find papers relating to Guided Imagery in Music – a specific approach utilised by music therapists involving receptive listening with the therapist guiding the client through images evoked.

Databases to search:

Allied & Complementary Medicine

Biblioline (RILM,RISM,)

British Nursing Index

CAIRSS

CENTRAL

CINAHL (R)

Cochrane Library

DH-DATA

Education abstracts

ERIC

LILACS

Medline

MTDATA 4

MT World online database

MT Temple University

OVID Gateway

Project MUSE

PsycINFO

PubMed

Social Sciences Abstracts

Social Work Abstracts

Sociological Abstracts

Web of Science

2. Following from a search of electronic databases, further searches to be

conducted by hand in each of the major music therapy journals:

Approaches

Arts and Health: An international journal for research, policy and practice

The Arts in Psychotherapy

Australian Journal of Music Therapy

International Journal of Arts Medicine

Journal of Music Therapy

British Journal of music therapy

Music and Arts in Action

Music and Medicine

Music Therapy Perspectives

Music Therapy Today

New Zealand Journal of Music Therapy

Canadian Journal of Music Therapy

Nordic Journal of Music Therapy

Voices: A World forum for music therapy

3. Reference searching: References of all identified studies to be inspected for more studies.

4. Personal contact: The contact authors of relevant reviews or studies to be contacted to enquire about other sources of relevant information.

5. Review articles: Inspect references of relevant review articles for further literature

6. Cited reference search (forward search): Search ISI web of science for articles citing any of the included studies, in order to identify any more recent studies that might have been missed.

7. Books and book chapters Guildhall School of Music & Drama

Nordoff-Robbins Library

British Library

8. Theses and dissertations musictherapyworld.de

Aalborg University

Grieg Academy Music Therapy Centre, Norway

Roehampton University (UK)

GSMD (UK)
University of West England (Bristol, UK)

Anglia Ruskin University(Cambridge, UK)

Nordoff-Robbins (London, UK)

Queen Mary University Edinburgh

Royal Academy of Music, Wales

Nordoff-Robbins Manchester

Temple University [www.temple.edu/musictherapy](http://www.temple.edu/musictherapy)

British Library Catalogue

US National Library of Medicine

9. Research Databases British Association for Music Therapy (BAMT) Research Network Database

10. Conference Proceedings World Federation of Music Therapy

European Music Therapy Confederation

International Music Therapy Associations

BIOSYS previews

ZETOC

Conference papers index

**Selection of studies:**

Detailed citations (title and abstract) will be screened independently by 1 researcher according to the above criteria and marked as include; exclude or uncertain. Twenty-five percent will be screened by a second researcher. All studies agreed not to meet inclusion will be excluded. Disagreements will be resolved first by discussion, and then by review by a third researcher if required. Full papers will then be retrieved, and those marked as uncertain reviewed against the inclusion criteria. Comparisons between the 2 researchers will again be made and areas of disagreement, or any further papers marked as uncertain will be discussed and resolved as outlined above. Authors will be contacted for further information, should this be required.

**Quality Assessment:**

It is likely that the majority of papers will be observational, case reports or theoretical and therefore likely to have a high level of bias. Given the variation in music therapy training, models and approaches, these variables will be taken into account as part of the review and papers rated using the EPPI approach (trustworthiness; appropriateness in answering review question; study relevance; overall weight of evidence provided). For prospective studies, study design, allocation and concealment will be noted during data extraction and outcomes analysed according to study quality.Twenty-five percent of papers will be quality assessed by a second researcher. Disagreements will be discussed and resolved with a 3^rd^ researcher if necessary.

**Data Extraction:**

Literature searches and citations will be managed and saved using Reference Manager software. The following categories will be used on a data collection proforma and entered independently by 1 researcher into an excel database. The proforma will be piloted with 10 studies and amended accordingly. 25% of the papers will be checked for accuracy of coding by a second researcher. Disagreements will be discussed and resolved with a 3^rd^ researcher if necessary.

**Data Collection Proforma:**

**Reference:** Author/Date/Title/Journal/Source

**Included:** Y/N

**Reason for exclusion:** Frequency less than twice a week/ Not psychiatric disorder/ not music therapy

**Type of paper:** Journal/Book chapter/ Conference/ Dissertation/ Unpublished

**Research Method/Design**

Randomised Controlled trials, Case Controlled Trials, Clinical non-controlled trials, Qualitative Research, Clinical Protocols, Case Studies, Theoretical Papers with Case examples, Theoretical Papers, Expert opinion

(from Odell-Miller, 2007)

**Country:**

**Diagnoses and criterion used:** ICD/DSM categories

**Setting:**

Inpatient /Outpatient /Community (non-health care provider eg. charity)

**Group/individual therapy:**

**Frequency of therapy:**

**Number of sessions:** Offered/Attended

**Duration of therapy:**

**Music Therapy Approaches Used:**

Active/Receptive/Mixed active and receptive

Structured/Unstructured

Pre-composed/Structured improvisation/Free improvisation

Focus of therapeutic attention/Aims of therapy

Description of techniques and interventions

**Theories informing approach:**

Psychodynamic/Psychoanalytic/Humanistic/Neuroscientific/Psychology

**Adaptations specific for increased frequency of therapy:**

**Reported experiences:** Positive effects/Negative effects reported for each approach

**Prospective study results:**

**Allocation:** Randomised/Quasi-randomised/Observational

**Allocation concealment:** Y/N/Not reported

**Blinding:** Blind/Double-blind/None/Not reported

**Demographics:** N/ age range and mean/ gender %

**Interventions:** Music therapy/ Details of additional treatment

**Comparison:** Details/N

**Attrition:** Number dropped out/%

**Outcome measures and results:** Outcome/Scale/Results

**Other notes:**

**Data Synthesis:**

Narrative synthesis will be performed for objectives 1 and 2; Quantitative outcomes will be reported for objective 3, and will be subject to content analysis. Narrative synthesis will be based upon current guidelines (Popay, Rodgers, Sowden, Petticrew, Britten, Rodgers et al., 2006; Rodgers, Sowden, Petticrew, Arai, Roberts, Britten et al., 2009) and will broadly follow steps of:

1. Developing a preliminary synthesis

Study characteristics and results will be tabulated. Approaches, theories and outcomes (positive and negative) will be categorized with descriptions. Studies will be grouped by diagnosis, theory, intervention type, design and outcomes. Approaches with similar outcomes and underlying theories will be integrated and subject to thematic analysis.

1. Exploring relationships within and between studies

Relationships will be explored by tabulation of studies, approaches and outcomes with analyses of sub-groups. A conceptual map will be drawn up to link approaches, proposed processes and outcomes and summarise the synthesis.

1. Assessing the robustness of the synthesis product

Quality assessment, critical reflection on the synthesis process and peer review with music therapists and researchers will be used.

**References:**

Drieschner K, & Pioch A. (2002). Therapeutic methods of experienced music therapists as a function of the kind of clients and the goals of therapy. *Music Therapy Today*, October, available at <http://www.mttd.com/modules/mmmagazine/issues/20021018120155/20021018120645/DrieschnerMTT.pdf>

Gold C, Dahle T, Heldal TO & Wigram T (2006). Music therapy for people with schizophrenia or other psychoses: a systematic review and meta-analysis. *British Journal of Music Therapy* 20 (2), pp.100-108.

Gold C, Solli HP, Krüger V & Lie SA (2009). Dose-Response relationship in music therapy for people with serious mental disorders: Systematic review and meta-analysis. *Clinical Psychology Review* 29 pp.193-207.

National Collaborating Centre for Mental Health (NCCMH), (2010). *Schizophrenia: The NICE Guideline on Core Interventions in the Treatment and Management of Schizophrenia in Adults in Primary and Secondary Care: Updated Edition.* National Clinical Guideline Number 82. London: The British Psychological Society & The Royal College of Psychiatrists

Odell-Miller, H. (2007). The practice of music therapy for adults with mental health problems: The relationship between diagnosis and clinical method. PhD Thesis, Aalborg: Aalborg University

Popay, J., Roberts, H., Sowden, A., Petticrew, M., Arai, L., Britten, N., Rodgers, M., Roen, K. & Duffy, S. (2006). *Guidance on the Conduct of Narrative Synthesis in Systematic Reviews: Final Report.* Swindon: ESRC Methods Programme.

Rodgers, M., Sowden, A., Petticrew, M., Arai, L., Roberts, H., Britten, N. & Popay, J. (2009). Testing methodological guidance on the conduct of narrative synthesis in systematic reviews: Effectiveness of interventions to promote smoke alarm ownership and function. *Evaluation15* (1), 47-71.

Silverman, M (2003). The influence of music on the symptoms of psychosis: A meta-analysis. *Journal of Music Therapy* 40: 27-40.
