# Supplementary material for: A Systematic Review of Music Therapy Practice and Outcomes with Acute Adult Psychiatric In-Patients
Source: PLoS One. 2013 Aug 2;8(8):e70252. doi: 10.1371/journal.pone.0070252 (PMC3732280; doi:10.1371/journal.pone.0070252)
Supplement: Information S2 — Search sources and example of search strategy. (DOCX) [file pone.0070252.s002.docx]

**Supporting information S2: Search sources for systematic reviews and example search strategy**

Databases: AMED, Biblioline (RILM, RISM), British Nursing Index, CAIRSS, CENTRAL, CINAHL (R), Cochrane Library, DH-DATA, Education abstracts, ERIC, LILACS, Medline, MTDATA 4, Music Therapy World online database, Temple University Music Therapy Database, OVID Gateway, Project MUSE, PsycINFO, Pubmed, Social Sciences Abstracts, Social Work Abstracts, Sociological Abstracts, and Web of Science.

Journals that were hand searched included: *Approaches, Arts and Health: An International Journal for Research, Policy and Practice, The Arts in Psychotherapy, International Journal of Arts Medicine, Australian Journal of Music Therapy, Journal of Music Therapy, British Journal of music therapy, Music and Arts in Action, Music and Medicine, Music Therapy Perspectives, Music Therapy Today, Musik Therapeutische Umschau, Musikterapi, Musikterapi in Psychiatrie, New Zealand Journal of Music Therapy, Canadian Journal of Music Therapy, Nordic Journal of Music Therapy and Voices: A world forum for music therapy*.

Library catalogues: Anglia Ruskin University, British Library, Guildhall School of Music & Drama, Nordoff-Robbins, Queen Mary University Edinburgh, Roehampton, Royal Academy of Music, Wales, University of West England, ZETOC

Music therapy research databases: British Association for Music Therapy, Nordoff-Robbins

International indexes of theses and dissertations: musictherapyworld.de, Aalborg University, Grieg Academy Music Therapy Centre Norway, Temple University

Conference proceedings: were identified from indexes of the World Federation of Music Therapy, European Music Therapy Confederation, International music therapy associations, BIOSYS previews, ZETOC and the Conference papers index.

| **Date** | **Provider** | **Database** | **Initial results** |
| --- | --- | --- | --- |
| **17.02.11** | **Ovid** | AMED, 1985-2011 | 285 |
|  |  | Embase classic + Embase, 1947-2011 | 8902 |
|  |  | HMIC, 1983-2011 | 185 |
|  |  | Medline, 1948-2011 | 7280 |
|  |  | Medline in process | 108 |
|  |  | Ovid Books | 24 |
|  | **Ebsco Host/Biblioline** | RILM, 1835-2011 | 1598 |
|  |  | RISM, 1850-2011 | 4 |
|  |  | CINAHL+, 1937-2011 | 846 |
|  |  | Psychinfo, 1800-2011 | 12068 |
|  |  | Psycarticles, 1894-2011 | 491 |
| **18.02.11** | **Trinity Western University** | CAIRSS for Music | 526 |
| **21.02.11** | **Cochrane Collaboration** | Cochrane Library, 1898-2011 | 671 |
|  | **Informaworld** | Education Research Abstracts, 1995-2011 | 20 |
|  |  | ERIC, 1966-2011 | 1109 |
|  | **Virtual Health Library** | LILACS | 97 |
|  | **Music therapy world**  **2001-2008** | Papers | 10 |
|  | **www.musictherapyworld.de** | Conferences | 2 |
|  |  | Dissertations | 20 |
|  | **Temple University** | Archives of Helen Bonny | 5 |
|  |  | Archives of Mary Priestley | 0 |
|  |  | Database of journals and books, 1947-2009 | 3708 |
|  |  | Project Muse, 1995-2011 | 5 |
| **22.02.11** | **ISI Web of Knowledge** | Social Science Abstracts | 135 |
|  |  | Web of science, 1970-2011 | 585 |
|  | **Proquest/CSA** | Social services abstracts, 1979-2011 and sociological abstracts, 1952-2011 | 2083 |
| **24.02.11** |  | British Nursing Index, 1993-2011 | 71 |
|  |  | British Nursing archive, 1985-1996 | 21 |
|  | **Elsevier** | Science direct, 1823-2011 | 1225 |
|  | **EBSCO Host** | Music Index, 1970-2011 | 1287 |
|  |  | **TOTAL** | 43371 |
|  |  | **After removal of duplicates** | 15990 |

**Example search: Ebsco Host (RILM, RISM, CINAHL+, Psycinfo, Psycharticles)**

Boolean/Phrase search:

( * musi* OR musi* OR * sound* OR sound* OR * acou OR acou* OR gim ) AND ( psychiatr* OR mental* or schizophrenia OR psychosis OR psychotic )

In all available fields.
