# Supplementary material for: A Systematic Review of Music Therapy Practice and Outcomes with Acute Adult Psychiatric In-Patients
Source: PLoS One. 2013 Aug 2;8(8):e70252. doi: 10.1371/journal.pone.0070252 (PMC3732280; doi:10.1371/journal.pone.0070252)
Supplement: Information S3 — Data extraction form. (DOC) [file pone.0070252.s003.doc]

**Supporting Information 3. Data Extraction Fields and definitions.**

Data were extracted into an excel database with the following fields:

| **EXCEL WORKSHEET 1** | **Paper description, service description and client and setting observations** |
| --- | --- |
| **Included** | Y- Yes: N- No |
| **Reason for exclusion: 1.Not acute mental health inpatients 2.Not Music Therapy** | Exclude if: 1.Setting is not for acute psychiatric inpatients (eg. day hospital, rehab) 2. Exclude if music is used primarily for background music, or without a therapeutic relationship ie. the therapist does not engage with the patient therapeutically before, during or after musical experience. |
| **Publication type** | Journal/Conference Proceeding/ Dissertation/ Book (chapter) |
| **Paper type** | Theoretical clinical, Theoretical clinical with case examples, Case Study, Case Series, Research, Clinical Manual, Literature Review |
| **Country** | Country/ Countries of origin |
| **Diagnoses and criterion used** | List any diagnoses treated, along with criteria (ICD/DSM) if listed. List any other criteria used for inclusion or exclusion to music therapy (eg. high/low functioning, acuteness of symptoms) |
| **Setting** | Note the setting and any details regarding size, purpose eg. number of beds/wards, average length of stay. |
| **Gp/Ind** | Note whether group, individual or a mixture are used in music therapy. Note whether the author defines any considerations as to which modality is used. |
| **Frequency** | Frequency of sessions per week |
| **Number of sessions offered** | Number of sessions offered to a patient during their stay (if specified in case study/research) |
| **Number of sessions attended** | Number of sessions attended by patient (if specified in case study/research) |
| **Length of session** | Length of the session in minutes |
| **Duration of therapy** | Any observations as to how long patients attend music therapy (eg. single session, 3 weeks). Note if any aspect of this is on an outpatient basis. |
| **Music Therapy Approaches Used: 1.Act/Rec 2.Struct/Unstructured 3.Precomp-Improv** | 1. Degree to which active music making and receptive listening are used in music therapy. 2. Active/Receptive/Mixed (mostly active/mostly rec/equal) 3.Degree of structure provided by therapist in the session 4.Structured/Unstructured/Semi-structured (guided by therapist/guided by patients) 5.Degree to which precomposed music and improvisation are used. 6.Precomposed only/Improvisation only/ Mixed (mostly precomposed/mostly improvisation) |
| **Rationale** | Reasons provided for the use of music therapy with this client group. Note any key theories that are referenced along with author (eg. Psychoanalytic- Freud; Inpatient Psychotherapy- Yalom). |
| **Therapy Aims** | Aims of music therapy specified by author |
| **Referral Criteria** | Any criteria used for referring patients to music therapy (indications/contraindications) |
| **Techniques/Interventions** | Detail as to how music therapy is delivered by the therapist within the sessions. |
| **Adaptations/Observations to:1. Setting 2.Client Group** | Note any key adaptations used by the therapist or any observations noted by the author that are particular to the setting (short-term hospitals) or client group (acute symptomatology). |
| **Reported Experiences: 1.Positive 2.Negative** | Note any positive or negative experiences provided by the author. |
| **Other Notes** | Any thoughts that may occur, questions or observations of interest. |

| **Excel Worksheet 2:** | **Research Papers** |
| --- | --- |
| **Study Aims** | Aims and objectives of study |
| **Hypothesis** | Insert only if stated explicitly in paper |
| **Predictor Variables** | Any variables thought to predict outcome variables (independent variables) |
| **Outcome Variables** | Any variables thought to change as a function of changes in the predictor variable (dependent variables) |
| **Process Variables** | Any other variables measured relating to processes of intervention |
| **Measures** | Any questionnaires, rating scales used to assess change. Note if the measure is an established scale or designed by the researcher. Detail of interviews etc. |
| **Method** | Research method eg. Randomised Controlled Trial, Case Controlled Trial, Clinical non-controlled trials, Qualitative research, Clinical Protocol |
| **Inclusion and Exclusion Criteria** | Any inclusion or exclusion criteria stated by author. |
| **Sample Size** | Number of participants in study, number in each arm of study if applicable |
| **Study Results** | Numerical results for quantitative studies. For qualitative studies, list key themes or points arising from research. |
| **Limitations** | Any limitations as observed by the author of the paper |
| **Other Notes** | Your own thoughts on the study- any further limitations not mentioned in paper. |

| **Excel Worksheet 3:** | **Music therapist approaches and techniques** |
| --- | --- |
| **Approach** | Note if a particular school of music therapy or thought is mentioned eg. Nordoff-Robbins, GIM, Analytic Music Therapy, Wheeler’s levels of music therapy, Yalom’s inpatient Psychotherapy, CBT. |
| **L1/L2/L3** | Mark if the author refers to any of the 3 levels as defined by Wheeler |
| **Function Level** | Note if the author provides groups for different levels of functioning. |
| **Therapist Leadership: 1.Active (Directive) 2.Non-directive** | Level of direction by therapist during the group. Ranging from:Active (directive), Active with loose structure, Non-directive (guided fully by patients) |
| **Session Structure: 1.Opening/Closing 2.Musical Events** | 1.Note if the session has an opening and or closing section and describe what is done. 2.Note any structuring of musical events and how the therapist does this. |
| **Active:** | Mark if any of these approaches are used within sessions. |
| **Improvisation: 1.Free/Associative with verbal processing 2.Thematic 3.Degree of control** | 1.Free improvisation, with no structure 2. Improvisation on a given theme eg. ‘anger’ or ‘the sea’ 3.Improvisation with some rules provided eg. one member leads the group, all to play quietly |
| **Recreative: 1.Musical performance ensembles 2.Group singing 3.Rhythmic activities 4.Song/Music Reception** | 1.Patients learn and perform precomposed music 2.Patients sing or play precomposed music 3.Structured rhythmic activities to precomposed music 4.Patients listen to music performed to them by another person |
| **Composition: 1.Song-writing 2.Musical composition** | 1.Patients compose a song by writing lyrics and/or music 2.Patients compose a piece of music |
| **Receptive: 1.Relaxation to taped music 2.Creative movement to taped music 3.Playback of taped improvisation 4.Lyric Analysis 5.Music/song reminiscence 6.Music/Song collage 7.Music selection with group discussion 8.Structured affective listening 9.GIM 10.Music Games** | 1. Patients listen to pre-recorded music and are guided in relaxation exercises 2.Patients are guided in moving to pre-recorded music 3.Patients listen back to a recording of the improvisation that they have just played 4.Patients analyse the lyrics to a popular song 5.Patients listen to music with the aim of evoking memories associated with that particular piece of music 6.Patients choose music that is meaningful to them and are assisted in making a tape/playlist of songs 7.Patients take turns to choose a piece of music to listen to and then discuss the music afterwards 8.Music is chosen to represent a specific emotion and then used as a stimulus for group discussion 9.The patient listens to a pre-selected program of music and is guided by therapist in discussing the images evoked. 10.Games played with music focus eg. musical bingo, musical charades |
| **Use of art/ poetry/ dance/ movement** | Note if other art forms are used in conjunction with music eg. drawing, poetry writing, movement, dance |
| **Use of words** | Note whether the therapist uses verbal discussion in the sessions and the extent to which this is used. |
| **Didactic/ musical instruction** | Tuition of music as a part of therapy eg. learning the guitar, learning music theory. |
