# Supplementary material for: A Systematic Review of Music Therapy Practice and Outcomes with Acute Adult Psychiatric In-Patients
Source: PLoS One. 2013 Aug 2;8(8):e70252. doi: 10.1371/journal.pone.0070252 (PMC3732280; doi:10.1371/journal.pone.0070252)
Supplement: Information S4 — Paper characteristics. (DOCX) [file pone.0070252.s004.docx]

| **Supplementary Information 4: Paper Characteristics of included papers by country** | | | | | | | | **Individual** |  | **Group** |  |  |  |  |  |
| --- | --- | --- | --- | --- | --- | --- | --- | --- | --- | --- | --- | --- | --- | --- | --- |
| **Reference** | **Paper type** | **Country** | **Quality (WoED)** | **Diagnosis** | **Duration of therapy** | **Duration of inpatient stay** | **N Sessions attended** | **Freq** | **Length of session** | **Freq per week** | **Length of session** | **Format** | **Location** | **Gp Size** | **Co-therapist (Yes/No)** |
| **Clemencic-Jones (1998)^38^** | Research-Pilot evaluation | Australia | 0.62 | Mixed | 6 week pilot | ns | 6 sessions offered: 4/6 attended more than 3 sessions; 2/6 only 1 session. | x | x | 1pw | 30-60mins | Closed | on ward | 3 - 6 | Y |
| **Cullen (1993)^39^** | Theoretical Clinical case vignettes | Australia | 0.77 | Mixed | 2-4 weeks | 2-4 weeks | ns | x | x | 2pw | 60mins | open | on ward | ns | N |
| **Dye (1994)^44^** | Research- Piloting of client evaluation of music therapy | Australia | 0.63 | Mixed | 1 month | ns | 9 | x | x | 1pw | 60mins | ns | ns | Listening: 3 - 6 Singing: 8 | N |
| **Featherstone (2008)^47^** | Pilot service evaluation | Australia | 0.39 | Mixed | 5 weeks | 2 weeks | 3 attended all 10 sessions; 1 attended 5; 1 attended 7; 3 attended 2-4 sessions; 1 dropped out before attending; 1 attended 1 session. 3 inpatients came later for one-off sessions; 2 joined later (in first 2 weeks) | x | x | 2pw | ns | Closed | off ward | 7 - 12 | Y |
| **Morgan, Bartrop, Telfer & Tennant (2011)^74^** | Research Quasi-RCT | Australia | 0.87 | DSM(IV) schizophrenia, schizoaffective disorder or BPAD. | 2 weeks | ns | 4 sessions: Non-completers: 5 Tx, 6 ctl Loss to followup:12 Tx, 11 ctl | 2pw | 10-30mins | x | x | x | x | x | X |
| **Storz (2005)^112^** | Theoretical-Clinical | Austria | 0.57 | Psychosis and Personality Disorders | 10-25 sessions | 2-4 months | 10 to 25 | ns | 60mins | ns | 60mins | ns | ns | ns | N |
| De Backer & Van Camp (2003)^41^ | Case study | Belgium | 0.89 | Adjustment disorder ICD 10: 309.28: | 12 months:Gp:8mthInd:4mth | ns | Approx 80 | 1pw | 45mins | 2pw | 45mins | ns | off ward | ns | ns |
| De Backer, J. (2006)^10^ | Research- Single case design | Belgium | 0.90 | Psychosis ICD 20-29 | 3-6 months | ns | ns | 1pw | 45mins | 2pw | 45mins | Semi-open | off ward | ns | N |
| Moura Costa & Negreiros Vianna (2011)^77^ | Clinical theoretical | Brazil | 0.66 | Psychosis | na | ns | ns | x | x | 2-3pw | 60-90mins | Semi-open | ns | 8 - 12 | Y |
| **Arnason (1993)^29^** | Theoretical Clinical case vignettes | Canada | 1.00 | Mixed | May only attend 1 session due to short stay | ns - notes short stay | ns | x | x | ns | ns | Semi-open | on ward | ns | Y |
| Bonde, Hannibal. & Pedersen (2012)^32^ | Survey | Denmark | 0.60 | Mostly schizophrenia/psychosis and personality disorders. Also treat mood disorders. Rarely treat substance misuse and ED. | ns | ns | ns | ns | ns | ns | ns | ns | ns | ns | ns |
| Frederiksen & Lindvang (1998)^49^ | Research- small survey of 5 therapists | Denmark | 0.63 | Mixed mostly schizophrenia, BPAD, BPD, depression | ns | ns | ns | 1pw | 30-45mins | 1-2pw | 45mins | Open and closed groups | on and off ward groups | 4 | Y |
| Hannibal (2002)^56^ | Clinical theoretical | Denmark | 1.00 | Mixed | ns | ns | 3 assessments | ns | ns | x | x | x | x | x | X |
| Hannibal (2005)^57^ | Research- Service evaluation | Denmark | 0.74 | Mixed mostly schizophrenia and personality disorder | Average 14 sessions | ns | Average 17.44 sessions offered. Average attendance 14 sessions (cancelled 3.45 sessions) - 20% cancellation rate | ns | ns | ns | ns | ns | ns | ns | ns |
| Hannibal, Pedersen, Hestbaek, Sorensen & Munk-Jorgensen (2012)^58^ | Research- observational | Denmark | 0.73 | Schizophrenia (F2) or Personality Disorder (F6) | ns | ns | Average 18 (sd 5.5) | ns | ns | ns | ns | ns | ns | 1.PD: 5-7 2.Sz: >5 | ns |
| Jensen (2000)^63^ | Clinical theoretical with case example | Denmark | 0.89 | Mixed | ns- often short | Short stay | ns | x | x | 2pw | 45mins | Open | on ward | ns | ns |
| Jensen (2002)^64^ | Clinical theoretical | Denmark | 0.89 | Mixed | ns | ns | ns | ns | ns | x | x | x | x | x | x |
| **Lindvang & Frederiksen (2008)^66^** | Clinical theoretical | Denmark | 1.00 | Mixed | ns- often short | 1-2 weeks | Mainly 1-2 (3-22) | x | x | 2pw | 45mins | Open | on ward | 3 - 5 | Y |
| Lindvang (2005)^67^ | Case study | Denmark | 0.89 | Schizophrenia | 6 months | Began as inpatient for a few weeks then outpatient | 20 | 1pw | 60mins | x | x | x | x | x | x |
| Lund (2008)^68^ | Clinical theoretical | Denmark | 0.93 | Mixed | na | ns | 6-8 sessions | x | x | 1pw | 60mins | Open and closed groups | on and off ward groups | 8 | ns |
| Moe (2002)^72^ | Clinical theoretical based on PhD research | Denmark | 0.89 | Schizophrenia | 6 months | ns | ns | x | x | ns | 90mins | Slow open | ns | 3 - 5 | N |
| Moe, Roesen & Raben (2000)^73^ | Research- Observational quantitative | Denmark | 0.67 | Schizophrenia and schizotypal disorders (F20-29) | 6 months | ns | 23-32 | x | x | 1-2pw | 90mins | Slow open | off ward | 3 - 5 | N |
| **Abs (1983)^27^** | Clinical theoretical | Germany | 0.63 | Mixed | ns | ns | ns | 1pw | ns | ns | ns | ns | ns | ns | ns |
| **Baumgarten & Mahns (1986)^30^** | Case study | Germany | 0.77 | Mixed | 10 weeks | ns | S-3=3 S4=4 S5=3 S6=4 S7=3; S8=3 S9=3 S10=3 | ns | ns | 1pw | 60mins | semi-closed | off ward | 4 | Y |
| Exner (1998) ^45^ | Case study | Germany | 0.82 | Schizophreniform psychosis | 2 years- began as acute inpatient. Duration of acute phase not specified. | ns | Approx 104 | 1pw | 60mins | x | x | x | x | x | X |
| **Haase & Reinhardt (2011)^35^** | Case study | Germany | 0.82 | Anxious-Avoidant personality disorder | 6 months | ns | ns | ns | ns | ns | ns | ns | ns | 30-40 | N |
| **Hopster (2005)^61^** | Research - observational | Germany | 0.76 | Mixed | Around half of patients treated for 4 weeks or less | <4wks | ns | ns | ns | x | x | x | x | x | X |
| **Maler, von Wietersheim, Schurbohm, & Nagel (1994)^69^** | Theoretical Clinical/Research | Germany | 0.57 | Mixed | 3 weeks | ns | 10 sessions offered | x | x | 3pw | 90 minutes | semi-closed | ns | 6 | N |
| **Metzner (2003)^71^** | Case study | Germany | 0.93 | Acute psychotic condition | 2.5 months | ns | 5 | ns | ns | x | x | x | x | x | x |
| Metzner (2010)^72^ | Case study | Germany | 0.83 | Chronic paranoid-hallucinatory psychosis with secondary addiction disorder (alcohol and drugs) | Almost 1 year | Almost 1 year, length of acute phase not specified | 78 | 2pw | 30mins | x | x | x | x | x | x |
| **Reker (1991)^90^** | Research - observational | Germany | 0.60 | Schizophrenia | Up to 38 weeks | 19.4wks (3-75wks) | 3-38 sessions (average 9) | x | x | 1pw | 60mins | Open | off ward | 5 - 7 | N |
| Seitz (2002)^93^ | Theoretical with case examples | Germany | 0.59 | Psychosis | ns | ns | na | ns | ns | ns | ns | ns | ns | ns | ns |
| Strehlow & Piegler (2011)^113^ | Theoretical Clinical | Germany | 1.00 | Mixed Case study: Personality Disorder | ns | ns | ns | 2pw | 30mins | 2pw | 75mins | Slow open | off ward | 6 | N |
| **Strunck (1986)^114^** | Theoretical - Clinical | Germany | 0.70 | Mixed neurosis, psychosis, substance abuse | ns | ns | 2 attended of 3 offered | ns | ns | ns | ns | open | on ward | ns | N |
| **Ulrich, Houtmans & Gold (2007)^118^** | Research-RCT | Germany | 0.90 | Schizophrenia ICD F20-29 | 5 weeks | ns | Average 7.5 sessions attended. 10 sessions offered | x | x | 2pw | 45mins | Semi-open | off ward | ns | N |
| Vogt-Schaeffer (1991)^119^ | Theoretical Clinical | Germany | 0.52 | Mixed | ns | ns | ns | na | na | na | na | na | na | na | na |
| Mössler, Fuchs, Heldal, Karterud, Kenner, Naesheim & Gold (2011)^76^ | Research summary with case vignettes | Germany, Austria, Norway, Australia | 0.92 | Mixed | 3 months | ns | 1. 24/24 2.25/25 3. 16/possible 24 (estimate) 4. 6/6 or 12 over 6 wks | 2pw | 45-50mins | x | x | x | x | x | X |
| **Sekeles (1999)^94^** | Clinical theoretical with case example | Israel | 0.89 | Pathological mourning | 6 months | 5 months | Approx 96 | 4pw | 60mins | x | x | x | x | x | X |
| **di Massimo, Boggio, D'Ulisse, Ferrara & Ordine (1998)^42^** | Theoretical/Preliminary research (no results) | Italy | 0.26 | Mixed | Average 3-4 weeks | Average 3-4 weeks | 589pts over 77 sessions- average 7.5 pps | x | x | 1-2pw | 45-60mins | open | on ward | 7 - 8 | Y |
| **Saitoh (2011)^92^** | Case study | Japan | 0.29 | Borderline personality disorder | 6 months | 6 months | Approx 24 | x | x | Fortnightly | 60mins | Open | ns | ns | ns |
| Gold, Solli, Krüger & Lie (2009)^4^ | Research- Systematic review and meta-analysis | Norway |  | Mixed | 1-6 months | na | 1. median 8/12; 2. 7.5 3. 11.8/20 4. M11.8/15 5. 23-32 6. M35.8/40 | 1-6pw | 20-90mins | 1-6pw | 20-90mins | ns | ns | ns | ns |
| Rolvsjord (2010)^13^ | Case studies (thesis) | Norway | 1.00 | Mixed- 1. BPD impulsive 2. PTSD | 1. 9 months 2. 3 years | 1. 9 months 2. 6 months Length of acute phase not specified | 1. 35 2. 133 | 1pw sometimes more often | 45-60mins | x | x | x | x | x | x |
| **Solli (2003)^106^** | Theoretical Clinical | Norway | 1.00 | Mixed | Sessions seen as standalone | ns | ns | x | x | 1 | 60mins | Open | On ward | ns | Y |
| **Solli (2006)^107^** | Theoretical Clinical with case examples | Norway | 1.00 | Mixed- mostly schizophrenia and substance abuse | Sessions seen as standalone | ns | ns | 1-2pw | 60mins | 1pw | 60mins | Open | On ward | ns | ns |
| **Solli (2008)^109^** | Case study | Norway | 1.00 | Mixed CS: Schizophrenia | 7 months | 7 months, length of acute phase not specified | 28 | 1pw | 45-60mins | ns | ns | ns | ns | ns | ns |
| **Solli (2009)^109^** | Theoretical Clinical with case study | Norway | 1.00 | Mixed- Case= Paranoid schizophrenia | ns approx 11 months | 12 months, length of acute phase not specified | ns | 1pw | 10-60mins | 1 | 60mins | Open | On ward | ns | ns |
| Solli & Rolvjsord (2009)^110^ | Theoretical Clinical with case example | Norway | 1.00 | Psychosis | 7 months | 7 months, length of acute phase not specified | 28 | 1pw | 60mins | x | x | x | x | x | x |
| Stige (2011)^111^ | Case study | Norway | 0.82 | Depression | 2.5 years mostly outpatient | ns | 66 (4 ax) | 1pw | 60mins | x | x | x | x | x | x |
| **Leite (2008)^65^** | Theoretical Clinical | Portugal | 1.00 | Mixed | ns | ns | ns | x | x | ns | ns | open | ns | ns | N |
| Ansdell & Meehan (2010)^28^ | Research- Qualitative idiographic interviews | UK | 0.77 | Mixed | Study: Interviewed after 10 weeks | na | na | 1pw | ns | x | x | x | x | x | x |
| Bunt, Pike, & Wren (1987) ^34^ | Research-Pilot evaluation | UK | 0.58 | Mixed | 8 weeks | ns | 48 sessions offered: 6 attended 35/48 (73%); Non attendance 13/48 (27%) | x | x | 1pw | 60mins | Closed | off ward | 6 | Y |
| **Davies & Richards (1998)^40^** | Case study | UK | 1.00 | Mixed | Sessions seen as standalone | ns | ns | x | x | 1pw | 60mins | open | on ward | 5 - 10 | Y |
| Fenwick (1970)^48^ | Clinical theoretical | UK | 0.52 | Mixed | ns | ns | ns | 1pw | 60mins | 1pw | 60mins | Open | on and off ward groups | ns | ns |
| **Gibson, Novakovic & Francis (2008)^50^** | Research-Service Evaluation | UK | 0.50 | Mixed | Study: 32 weeks | ns | ns | x | x | 1pw | ns | ns | on ward | ns | Y |
| **Grandison (1991)^54^** | Service evaluation | UK | 0.70 | Mixed | ns | 26 days | ns | 1-2pw | ns | 1pw | ns | open | on ward | 4 | N |
| Moss (1999)^75^ | Research- Pilot project | UK | 0.59 | Mixed | ns | ns | ns | ns | ns | ns | ns | ns | ns | 5 | N |
| Odell-Miller (1986)^82^ | Report | UK | 0.66 | Mixed | ns | ns | ns | x | x | ns | ns | ns | ns | ns | ns |
| Odell-Miller (1992)^12^ | Clinical theoretical | UK | 0.66 | Mixed | ns | ns | ns | ns | ns | 3pw | ns | ns | ns | ns | ns |
| Odell-Miller (2001)^83^ | Research- Rationale for study/protocol | UK |  | Mixed | 6 months | ns | na | ns | ns | ns | ns | ns | ns | ns | ns |
| Odell-Miller, Hughes & Westacott (2006)^84^ | Research - Randomised controlled trial | UK | 0.83 | Mixed | 6 months | ns | ns | ns | ns | ns | ns | ns | ns | ns | ns |
| **Pavlicevic (1987)^85^** | Theoretical Clinical- 2 case vignettes | UK | 0.72 | Mixed | ns | na | na | na | na | na | na | na | na | na | na |
| Priestley (1975)^86^ | Theoretical with case examples | UK | 0.77 | Mixed | na | na | na | ns | ns | 0.5-2pw | ns | open | on and off ward groups | 3 - 18 | Y |
| **Procter (2002)^87^** | Clinical- theoretical | UK | 0.70 | Mixed | ns | ns | ns | ns | ns | ns | ns | Open | on ward | ns | ns |
| **Rowland & Read (2011)^91^** | Research- Pilot evaluation | UK | 0.48 | Mixed | 25 weeks | ns | Patient A: 16/19, G: 14/21. Ward 1: 3/5 Ward 2: 4/6 | x | x | 1pw | 60mins | Closed | on ward | 5 | N |
| **Sloboda (2008)^104^** | Clinical Theoretical with case studies | UK | 0.77 | Mixed | ns | Short stay | ns | x | x | 1pw | ns | open | on ward | 6 | Y |
| Talwar, Crawford, Maratos, Nur, McDermott & Procter (2006)^116^ | Research-RCT | UK | 0.87 | Schizophrenia ICD F20-29 | 12 weeks | ns | All attended at least 1 session; 22 attended at least 4 sessions (67%); 7 (21%) attended all 12 sessions. Median = 8 sessions. | 1pw | 45mins | x | x | x | x | x | x |
| Blake & Bishop (1994)^31^ | Clinical theoretical | USA | 0.89 | PTSD | 2 weeks | 2 weeks | Suggest minimum 3 | 2pw | ns | ns | ns | ns | ns | ns | ns |
| **Braswell, et al(1986)^33^** | Scale development | USA | 0.55 | Mixed | na | ns | na | na | na | na | na | na | na | na | na |
| Cassity & Cassity (1994)^36^ | Research - survey | USA | 0.57 | Mixed | ns | ns | ns | na | na | na | na | na | na | na | na |
| **Cassity & Cassity (2006)^37^** | Clinical manual | USA | 0.59 | Mixed | na | May range from 1-50 sessions | Suggest up to 20 | ns | ns | x | x | x | x | x | X |
| **Cassity (1976)^38^** | Research- Quasi-RCT | USA | 0.48 | Mixed | 2 weeks | ns | 10 sessions offered | x | x | 5pw | 60mins | Closed | ns | 7 | N |
| Dvorkin (2008)^43^ | Theoretical Clinical | USA | 0.93 | Mixed | ns | ns | na | x | x | ns | ns | ns | ns | ns | ns |
| Eyre (2011)^46^ | Case study | USA | 0.59 | Psychosis - with anorexia, 🡪dissociative identity disorder | 6months | Multiple admissions over 6 month period | ns | 1-3pw | 30mins | ns | ns | ns | ns | ns | ns |
| **Goldberg (1989)^51^** | Theoretical- clinical practice | USA | 1.00 | Mixed- primarily schizophrenia; major affective disorders with and without psychosis; BPD | 17 days (2 days - 2 months) | 17 days (2 days - 2 months) | ns | Scheduled frequently | ns | 5pw | ns | 1. open 2. focused | ns | ns | N |
| **Goldberg (1994)^52^** | Theoretical -clinical | USA | 1.00 | Mixed mostly psychosis | na | 17 days (5-30) | ns | x | x | 5pw | 15-30mins as part of 60min pth | semi-open | ns | 4 - 8 | N |
| **Goldberg, McNiel & Binder (1988)^53^** | Research-Mixed methods | USA | 0.84 | Mixed mostly psychosis | 17 days | 17 days | ns - 201 sessions studied | x | x | 5pw | 50mins | Semi-open | on ward | ns | N |
| **Hara (1999)^59^** | Theoretical- Clinical Practice | USA | 0.93 | Mixed | ns | ns - notes short stay | ns | x | x | ns | ns | ns | ns | ns | N |
| **Heaney (1992)^60^** | Research - survey | USA | 0.69 | Mixed | average 10 days | 21 days | na | ns | ns | ns | ns | ns | ns | ns | ns |
| Hudson Smith (1991)^62^ | Case study | USA | 0.83 | Mixed CS: Depression | 18 months - 3 x hospitalisations - individual MT 2xpw for 8 months between 2nd and 3rd hospitalisation. | Hospitalised1: 30 days 2: 4.5 mth 3: 3 weeks | ns | x | x | Daily | 60mins | semi-closed | off ward | ns | N |
| **Murphy (1991)^78^** | Case study | USA | 1.00 | Mixed CS: Major bipolar affective disorder | 3.5 months | 3.5 months | 23 | x | x | Range of groups provided over week | Open singout 45 mins | Open and closed groups | Open- dayroom; closed- off | ns | N |
| **Murphy (1992)^79^** | Theoretical Clinical- 2 case vignettes | USA | 1.00 | Mixed CS 1: Depression CS 2: Severe depression | ns | 2 months | na | 1-2pw | ns | 2-3pw | ns | Open and closed groups | on ward | 4 - 10 | N |
| **Nolan & Ierardi (2007)^80^** | Theoretical Clinical - Detailed approaches | USA | 1.00 | ns | na | na | ns | na | na | na | na | na | na | na | na |
| **Nolan (1991)^81^** | Case study | USA | 1.00 | Mixed CS: Schizophrenia | ns | 3 weeks | ns | ns | ns | 3pw | 60mins | ns | On ward | 4 | N |
| Ragland (1973)^88^ | Theoretical-clinical | USA | 0.42 | Mixed | ns | ns- short term | ns | x | x | 3pw | 60mins | 1. Open 2. Patient select closed | 1. On ward 2. Off ward | ns | ns |
| **Ready (2011)^89^** | Case study as part of doctoral research | USA | 0.31 | Psychosis | na | 9 weeks (3 wks acute) | na | 1-2pw | 30min | 5pw | 60mins | ns | On unit | ns | ns |
| **Shultis (1999)^95^** | Theoretical-clinical | USA | 0.69 | Mixed | Single session | ns- short term | 1 | Single session | ns | ns | ns | ns- 3 types of group activity offered | ns | ns | ns |
| **Silverman & Marcionetti (2004)^96^** | Research-pre-post | USA | 0.62 | Primary Axis I- schizophrenia, shizoaffective disorder, bipolar disorder, major depressive disorder, psychosis nos | Single session over 3 weeks | 1 day - 1 month | 8 sessions studied; single session focus | x | x | 2pw | 45mins | Open | ns | 3 - 15 | N |
| **Silverman (2003)^97^** | Case study | USA | 1.00 | Schizophrenia | 36 days | 40 days | 21-24 | x | x | 5pw | 45mins | Open | Activities room on unit | Up - 24 | Y |
| Silverman (2007)^98^ | Research - survey | USA | 0.66 | Mixed | ns | Typically 7 months | na | 1-6pw | 31-45mins | 1-6pw | 31-45mins+ | ns | ns | Most 5 - 8 (1 - >21) | 61.7% N |
| **Silverman (2009a)^99^** | Theoretical-clinical with case example | USA | 0.83 | Mixed. Case study: Depressive disorder | 3 days | 3 days | 5 | x | x | 5pw | 45mins | open | on ward | 3 - 26 | N- hospital staff present |
| **Silverman (2009b)^100^** | Research-pre-post | USA | 0.67 | Mixed | Single session over 5 months | 3-5 days | Single session focus: mean ppts per session = 4 vs 3.46 ctl. Ppts attended 28/32 sessions (87.5%). Data collected over 28 sessions (15 exp, 13 control). | *x* | *x* | 2pw- single session approach | ns | semi-open (IC for research) | activity room on unit | ns | N |
| **Silverman (2010)^101^** | Research-survey | USA | 0.81 | Mixed | 5 days | 2wks - a few months | 3/15 attended more than one session ie. 12/15 attended only one session. | x | x | 5pw | 45mins | open | off ward | ns | N |
| **Silverman (2011a)^102^** | Research- Randomised clinical effectiveness study | USA | 0.56 | Mixed | Single session | 3-7 days | 16 - assume single session accessed | x | x | 1pw | ns | Open | ns | ns | N |
| **Silverman (2011b)^103^** | Research- Pilot randomised controlled trial | USA | 0.53 | Mixed | Study 1: 4 weeks Study 2: Single session | ns | ns | x | x | 3pw | 30-45mins | Open | ns | Study 1: 1 - 3 Study 2: 2 - 6 | N |
| **Smith (1975)^105^** | Clinical theoretical | USA | 0.59 | Mixed | ns | 27 days | ns | ns | ns | ns suggests high intensity is required | ns | ns | ns | ns | Ns |
| **Sullivan (2003)^115^** | Theoretical-Clinical | USA | 0.63 | Mixed | ns | ns | ns | x | x | Several times per week | 45-60mins | Open: Varying focus eg. listening, songwriting, singing, drumming | On ward | ns | N |
| **Thomas (2007)^117^** | Theoretical-Clinical | USA | 0.83 | Mixed | Short stay | ns- short term | ns | x | x | ns | ns | 1. Open 2. Closed focussed groups | 1. On ward 2. Off ward | *ns* | *ns* |
| **Wolfe (1996)^120^** | Theoretical-Clinical | USA | 0.59 | Mixed | Short stay | ns- short term | ns | x | x | 5pw | ns | ns | ns | 6 - 9 | ns |

X – Not used; ns – not specified; na – not applicable; CS- Case study; Y – Yes; N - No
