# Supplementary material for: A Systematic Review of Music Therapy Practice and Outcomes with Acute Adult Psychiatric In-Patients
Source: PLoS One. 2013 Aug 2;8(8):e70252. doi: 10.1371/journal.pone.0070252 (PMC3732280; doi:10.1371/journal.pone.0070252)
Supplement: Information S5 — Coverage of themes. (DOCX) [file pone.0070252.s005.docx]

**Supplementary Information 5. Paper characteristics and coverage of themes***

| **Reference** | **Country** | **Clinical Aims** | | | | | | | | **Setting specific themes** | | | | | **Patient specific themes** | | | | |
| --- | --- | --- | --- | --- | --- | --- | --- | --- | --- | --- | --- | --- | --- | --- | --- | --- | --- | --- | --- |
|  |  | **A1** | **A2** | **A3** | **A4** | **A5** | **A6** | **A7** | **A8** | **S1** | **S2** | **S3** | **S4** | **S5** | **P1** | **P2** | **P3** | **P4** | **P5** |
| **Abs (1983) ^27^** | Germany | x |  | x | x | x | x |  |  | x | x |  |  |  | x |  | x |  |  |
| Ansdell & Meehan (2010) ^28^ | UK |  |  | x |  |  |  | x |  |  |  |  |  |  |  |  | x |  |  |
| **Arnason (1993) ^29^** | Canada | x | x | x |  |  |  | x | x | x | x | x | x | x | x | x | x | x | x |
| **Baumgarten & Mahns (1986)^30^** | Germany |  |  |  |  |  |  |  |  |  |  |  |  |  |  |  | x | x | x |
| Blake & Bishop (1994) ^31^ | USA |  | x | x |  | x | x | x |  |  |  |  |  | x |  |  |  |  | x |
| Bonde, Hannibal. & Pedersen (2012).^32^ | Denmark | x | x | x | x | x | x | x | x | x | x | x | x | x |  | x |  |  |  |
| **Braswell, Brooks, Decuir, Humphrey, Jacobs & Sutton (1986)^33^** | USA |  |  |  |  |  |  |  |  |  |  |  |  |  |  | x |  |  |  |
| Bunt, Pike, & Wren (1987)^34^ | UK | x | x | x | x | x |  | x |  |  |  |  |  | x |  | x |  |  |  |
| Cassity & Cassity (1994)^35^ | USA |  |  |  |  |  |  |  |  |  |  |  |  |  |  | x |  |  |  |
| **Cassity & Cassity (2006)^36^** | USA |  |  |  |  |  |  | x | x |  |  |  |  | x |  |  |  |  |  |
| **Cassity (1976)^37^** | USA |  | x |  |  |  |  |  | x |  |  |  |  |  |  | x |  |  |  |
| **Clemencic-Jones (1998)^38^** | Australia | x | x | x | x | x | x | x | x | x |  |  |  |  |  | x | x |  |  |
| **Cullen (1993)^39^** | Australia |  | x | x | x |  |  |  |  |  |  |  |  | x |  |  |  |  | x |
| **Davies & Richards (1998)^40^** | UK | x | x |  |  |  |  | x |  | x |  | x | x | x | x | x | x |  |  |
| De Backer & Van Camp (2003)^41^ | Belgium |  | x |  | x | x |  | x |  | x | x | x |  |  | x | x |  |  | x |
| De Backer, J. (2006) ^10^ | Denmark |  | x |  |  | x |  | x |  | x |  | x |  |  |  | x | x | x | x |
| Dvorkin (2008)^43^ | USA | x | x | x | x |  |  | x | x |  |  |  |  | x | x | x | x |  |  |
| **Dye (1994) ^44^** | Australia |  |  |  |  |  |  |  |  |  |  |  |  |  |  | x |  |  |  |
| Exner (1998)^45^ | Germany |  | x | x |  | x | x | x |  |  |  |  |  |  |  |  |  | x |  |
| Eyre (2011)^46^ | USA |  | x |  |  | x | x |  |  |  |  |  |  |  | x | x | x |  |  |
| **Featherstone (2008)^47^** | Australia | x | x |  |  |  |  | x |  | x |  |  |  | x | x |  |  |  | x |
| Fenwick (1970)^48^ | UK |  | x |  | x |  |  | x | x | x |  |  |  |  |  | x |  | x |  |
| Frederiksen & Lindvang (1998)^49^ | Denmark |  | x |  | x |  |  | x |  | x | x | x | x | x | x | x |  |  | x |
| **Gibson, Novakovic & Francis (2008)^50^** | UK | x |  |  |  |  |  |  | x |  |  | x |  |  |  |  |  |  |  |
| Gold, Solli, Kraeger & Lie (2009) ^4^ | Norway | x | x |  | x | x | x |  | x |  |  |  |  |  |  |  |  |  |  |
| **Goldberg (1989)^51^** | USA | x |  |  | x |  |  | x | x |  |  |  |  | x | x | x |  |  |  |
| **Goldberg (1994)^52^** | USA |  | x |  |  |  |  | x | x |  | x | x |  | x | x |  |  |  | x |
| **Goldberg, McNiel & Binder (1988)^53^** | USA | x |  |  |  |  |  | x | x |  |  |  | x | x |  |  |  |  |  |
| **Grandison (1991)^54^** | UK |  | x | x |  |  |  |  |  | x | x | x |  | x |  | x |  |  | x |
| **Haase & Reinhardt (2011)^55^** | Germany |  | x | x |  | x |  | x |  | x |  |  |  |  | x |  | x | x | x |
| Hannibal (2002)^56^ | Denmark |  | x | x |  | x |  |  |  | x |  | x |  |  | x |  |  | x | x |
| Hannibal (2005)^57^ | Denmark |  |  |  |  |  |  |  |  |  |  |  |  |  | x | x |  |  | x |
| Hannibal, Pedersen, Hestbaek, Sorensen & Munk-Jorgensen (2012)^58^ | Denmark |  |  |  |  |  |  |  |  |  |  | x |  |  |  |  |  |  | x |
| **Hara (1999)^59^** | USA |  | x | x | x |  |  | x |  |  |  |  | x | x | x |  | x | x | x |
| **Heaney (1992)^60^** | USA |  |  |  |  |  |  |  |  | x | x | x |  |  |  |  |  |  |  |
| **Hopster (2005)^61^** | Germany |  | x |  |  |  |  |  |  |  |  |  |  | x |  |  | x |  |  |
| Hudson Smith (1991)^62^ | USA |  | x | x |  |  |  |  |  |  |  |  |  |  | x |  | x | x | x |
| Jensen (2000)^63^ | Denmark | x | x | x | x |  |  | x | x | x |  |  |  | x | x | x |  |  | x |
| Jensen (2002)^64^ | Denmark | x |  | x | x | x |  | x | x |  |  |  |  | x | x |  | x |  | x |
| **Leite (2008)^65^** | Portugal |  |  | x | x | x |  | x | x | x | x |  | x | x | x | x | x |  | x |
| **Lindvang & Frederiksen (2008)^66^** | Denmark |  | x | x |  |  |  |  |  | x |  | x | x |  | x | x |  | x | x |
| Lindvang (2005)^67^ | Denmark |  |  |  |  |  |  |  |  |  |  | x | x |  |  |  |  |  |  |
| Lund (2008)^68^ | Demark |  | x | x |  | x |  | x |  |  | x | x | x |  |  | x |  |  |  |
| **Maler, von Wietersheim, Schurbohm, & Nagel (1994)^69^** | Germany |  |  | x | x | x |  | x |  |  |  | x |  |  |  |  |  |  |  |
| **Metzner (2003)^70^** | Germany |  | x |  |  | x | x |  |  |  |  | x |  |  | x |  |  |  | x |
| Metzner (2010)^71^ | Germany |  |  |  |  |  |  | x |  |  |  | x |  |  |  |  |  | x |  |
| Moe (2002)^72^ | Denmark |  | x |  |  | x |  | x |  |  |  |  |  |  | x |  |  |  |  |
| Moe, Roesen & Raben (2000)^73^ | Denmark |  |  |  |  | x |  | x |  |  |  |  |  | x | x | x |  |  | x |
| **Morgan, Bartrop, Telfer & Tennant (2011)^74^** | Australia |  | x | x | x | x | x |  |  |  |  |  |  | x | x |  |  |  | x |
| Moss (1999)^75^ | UK | x | x | x | x |  | x |  |  |  |  | x |  |  |  |  |  |  |  |
| Mossler, Fuchs, Heldal, Karterud, Kenner, Naesheim & Gold (2011)^76^ | Multicentre European |  |  |  |  |  |  | x |  |  |  |  | x | x | x | x |  | x | x |
| Moura Costa & Negreiros Vianna (2011)^77^ | Brazil | x | x | x |  | x | x | x |  |  |  |  |  | x | x |  |  |  | x |
| **Murphy (1991)^78^** | USA | x | x |  | x | x |  | x | x | x |  | x |  |  | x | x | x |  |  |
| **Murphy (1992)^79^** | USA | x | x | x |  |  |  | x | x | x | x |  | x | x | x | x |  |  | x |
| **Nolan & Ierardi (2007)^80^** | USA |  | x | x | x | x | x | x | x |  |  |  |  | x | x |  |  |  |  |
| **Nolan (1991)^81^** | USA |  |  |  | x |  |  | x | x |  |  |  |  |  |  |  | x |  | x |
| Odell-Miller (1986)^82^ | UK | x |  | x | x |  |  | x | x |  | x |  |  | x | x |  |  |  |  |
| Odell-Miller (1992) ^12^ | UK |  |  |  |  | x |  | x | x |  | x | x |  | x |  |  |  |  |  |
| Odell-Miller (2001)^83^ | UK |  |  |  |  |  | x | x |  |  | x |  |  |  |  |  |  |  |  |
| Odell-Miller, Hughes & Westacott (2006)^84^ | UK |  |  |  | x |  |  | x |  |  |  |  |  |  |  | x |  |  |  |
| **Pavlicevic (1987)^85^** | UK |  |  |  |  |  |  |  |  |  |  | x |  |  |  | x | x | x | x |
| Priestley (1975)^86^ | UK | x | x | x | x |  |  | x | x | x |  | x | x | x | x | x | x |  |  |
| **Procter (2002)^87^** | UK |  |  |  | x |  |  | x | x | x |  |  |  |  |  |  |  |  | x |
| Ragland (1973)^88^ | USA |  | x | x | x | x |  |  |  | x |  | x |  |  |  | x |  |  | x |
| **Ready (2011)^89^** | USA |  |  |  | x |  |  |  |  |  |  |  |  |  |  |  |  |  | x |
| **Reker (1991)^90^** | Germany | x | x | x | x | x |  | x |  |  |  | x |  |  | x | x | x | x |  |
| Rolvsjord (2010) ^13^ | Norway |  |  |  |  |  |  | x |  |  | x |  |  |  | x |  | x |  | x |
| **Rowland & Read (2011)^91^** | UK |  | x | x | x | x |  | x |  |  | x | x |  |  |  |  |  |  |  |
| Seitz (2002)^93^ | Germany |  |  |  |  |  |  |  |  |  |  |  |  |  | x |  |  |  |  |
| **Sekeles (1999)^94^** | Israel |  |  | x | x |  |  | x |  |  |  |  |  |  | x | x |  |  | x |
| **Shultis (1999)^95^** | USA |  |  | x | x |  | x | x |  |  |  |  |  |  | x | x |  |  |  |
| **Silverman & Marcionetti (2004)^96^** | USA |  |  | x | x |  | x | x | x |  |  |  |  | x |  |  |  |  | x |
| **Silverman (2003b)^97^** | USA |  | x | x |  |  |  |  |  | x |  | x |  | x | x |  |  |  | x |
| Silverman (2007)^98^ | USA | x | x | x | x | x | x | x | x | x | x | x |  |  |  |  |  |  |  |
| **Silverman (2009a)^99^** | USA |  |  | x |  |  |  | x |  |  |  |  |  | x |  |  |  | x | x |
| **Silverman (2009b)^100^** | USA | x |  |  |  |  |  |  | x | x |  | x | x | x |  |  | x | x | x |
| **Silverman (2010)^101^** | USA |  | x | x |  |  |  | x | x |  |  |  |  |  |  |  |  |  | x |
| **Silverman (2011a)^102^** | USA |  |  |  |  |  |  | x | x |  |  |  |  |  |  |  |  |  | x |
| **Silverman (2011b)^103^** | USA |  |  |  |  |  |  | x |  |  |  |  |  | x |  |  |  | x | x |
| **Sloboda (2008) ^104^** | UK |  |  |  | x |  |  | x |  |  | x | x | x |  |  |  |  |  | x |
| **Smith (1975)^105^** | USA | x |  | x | x | x |  | x |  |  |  |  |  | x |  |  |  |  |  |
| **Solli (2003)^106^** | Norway | x | x | x | x |  |  | x | x | x |  | x | x | x | x | x |  |  |  |
| **Solli (2006)^107^** | Norway | x | x | x | x |  |  | x |  |  |  |  |  |  |  |  |  |  |  |
| **Solli (2008)^108^** | Norway | x |  | x | x |  |  | x | x | x |  | x |  |  | x | x |  | x |  |
| **Solli (2009)^109^** | Norway | x | x | x | x |  |  | x | x | x | x | x | x | x | x | x | x | x | x |
| Solli & Rolvsjord (2009)^110^ | Norway | x | x | x | x |  |  | x |  |  |  | x |  |  | x |  |  |  | x |
| Stige (2011)^111^ | Norway | x | x | x | x |  | x | x |  | x |  |  |  |  | x |  |  | x | x |
| **Storz (2005)^112^** | Austria |  | x |  |  | x |  | x |  |  |  |  |  | x |  |  |  | x |  |
| Strehlow & Piegler (2011)^113^ | Germany |  | x | x | x | x | x | x |  |  |  | x |  |  | x |  |  |  |  |
| **Strunck (1986)^114^** | Germany |  | x | x | x |  |  | x |  |  |  |  |  | x |  | x |  |  | x |
| **Sullivan (2003)^115^** | USA | x |  |  |  |  |  | x | x |  |  |  |  |  |  | x |  |  | x |
| Talwar et al., (2006)^116^ | UK |  | x | x | x |  | x |  |  |  |  |  |  | x |  |  |  |  |  |
| **Thomas (2007)^117^** | USA | x | x |  | x | x | x |  | x | x | x |  | x | x | x |  | x |  | x |
| **Ulrich et al.,(2007)^118^** | Germany |  | x |  |  |  |  |  |  |  |  |  |  | x |  | x |  |  |  |
| Vogt-Schaeffer (1991)^119^ | Germany | x | x | x | x |  |  | x | x | x | x | x |  |  |  |  |  |  |  |
| **Wolfe (1996)^120^** | USA | x |  | x | x | x |  | x |  |  |  | x |  | x |  | x | x | x | x |
| **TOTAL** |  | 34 | 54 | 50 | 47 | 33 | 20 | 65 | 34 | 31 | 21 | 37 | 18 | 42 | 43 | 40 | 25 | 22 | 48 |

* Papers considering acute inpatient music therapy only are highlighted in bold

Clinical aims: A1 = Engagement, A2 = Interpersonal, A3 = Self-expression and communication, A4 = Emotional, A5 = Cognitive, A6 = Symptom specific, A7 = Building Resources, A8 = Issues arising from hospitalisation

Setting specific themes: S1 = Hospital environment, S2 = Institutional structure, S3 = Multidisciplinary team, S4 = Patient turnover, S5 = Short time frame

Patient specific themes: P1 = Symptom severity, P2 = Functioning level, P3 = Reaction to hospitalisation, P4 = Previous experiences of therapy, P5 = Patient engagement
