# Supplementary material for: A Systematic Review of Music Therapy Practice and Outcomes with Acute Adult Psychiatric In-Patients
Source: PLoS One. 2013 Aug 2;8(8):e70252. doi: 10.1371/journal.pone.0070252 (PMC3732280; doi:10.1371/journal.pone.0070252)
Supplement: Information S6 — Analysis of client and setting characteristics. (DOCX) [file pone.0070252.s006.docx]

**Supplementary Information 6. Analysis of key features of music therapy work in acute psychiatric inpatient settings**

| **Feature** | **Impact** | **Approaches taken** | **Papers** |
| --- | --- | --- | --- |
| **Hospital environment** | |  |  |
| Chaotic and unsettled environment | Engagement and participation of patients Patients may be early/late Group programmes subject to change at short notice. Group programmes inadequately coordinated. Music therapy session is delayed or interrupted Music played by patients disturbs others Events on ward can come into group. | Therapist brings music therapy onto ward and treats whole ward as a subgroup in open ward groups. Joins with competing stimuli eg. TV. Team must provide therapy that complements and supports patients in hospital. Environment must support music therapist in bringing and assisting clients to access sessions. Therapist holds group in day room for safety. Mood on ward is positively influenced by music therapy sessions. Therapist works with client playing in music therapy sessions. Boundaries: Environmental boundaries used to help patients feel safe and secure but must not exclude staff. Consistency of time and place of group. | Abs, 1993^27^ Bonde et al., 2012^32^ De Backer, 2006^10^ Featherstone, 2008^47^ Grandison, 1991^55^ Hannibal, 2002^57^ Leite, 2008^65^ Lindvang & Frederiksen, 2008^66^ Murphy, 1992^79^ Priestley, 1975^87^ Ragland, 1973^89^ Silverman, 2003^97^ Silverman, 2009b^101^ Solli, 2006^107^ Solli, 2008^108^ Solli, 2009^109^ Thomas, 2007^118^ Vogt-Schaeffer, 1991^119^ |
| Limited availability of staff and resources: Finance, Staff, Space | Availability, repair and maintenance of instruments Availability to escort patients to group Suitability and consistency of therapy location First encounter usually occurs outside of therapy room Arts therapists usually not ward based | Music therapy offered to all on unit Therapist meets patient in ward round or coffee break Communication with Multidisciplinary Team | Arnason, 1993^29^ Clemencic-Jones, 1998^38^ Davies & Richards, 1998^40^ Dye, 1994^44^ Frederiksen & Lindvang, 1998^49^ Haase & Reinhardt, 2011^55^ Heaney, 1992^60^ Jensen, 2002^64^ Leite, 2008^65^ Murphy, 1992^79^ Silverman, 2007^98^ Vogt-Schaeffer, 1991^119^ |
| **Institution Structure** | |  |  |
| Staff turnover | Lack of consistency in involvement and communication with staff. Relationships with patients and services disrupted. |  | Heaney, 1992^60^ Rowland & Reed, 2011^91^ Vogt-Schaeffer, 1991^119^ |
| Service hierarchy | Difficult for patients to see past hierarchical role Admission/Discharge dictated by Consultant | Increased Multidisciplinary team communication. Involvement of staff in music sessions to provide opportunity to see staff member in different environment | Abs, 1983^27^ Goldberg, 1994^52^ |
| Fit of music therapy service with institution structure | No onward referral at discharge Small service- Part time employment limits integration, therapist unable to attend ward rounds Lack of fit between theoretical models of treatment Lack of distinction between activity and psychotherapy Focus upon medication and not therapy | Staff communication, supervision, education and training by music therapist. Use of co-therapist with another member of multidisciplinary team. Team discussions to find and maintain a coherent model of work. Clear boundaries maintained for music therapy. | Bonde et al., 2001^32^ De Backer & van Camp, 2003^41^ Frederiksen & Lindvang, 1998^49^ Grandison, 1991^54^ Heaney, 1992^60^ Murphy, 1992^79^ Odell-Miller, 1986^82^ Odell-Miller, 1992^12^ Odell-Miller, 2001^83^ Sloboda, 2008^104^ Solli, 2009^110^ Thomas, 2007^117^ Vogt-Schaeffer, 1991^119^ |
| Highly structured routine: Other appointments, visitors, smoking breaks, leave, group programme | Competing activities limit attendance and blur confidentiality/therapeutic boundaries. Difficulty for staff and patients to differentiate between groups. Lack of integration between wards Limited time for music therapist to prepare for group Patient preoccupation with leave and other hospital matters | Patients learn from other patients about music therapy service. | Abs, 1983^27^ Arnason, 1993^29^ Bunt, Pike & Wren, 1987^34^ Cullen, 1993,^39^ Davies & Richards, 1998^40^ Fenwick, 1970^48^ Goldberg, 1994^52^ Leite, 2008^65^ Lund, 2008^68^ Ragland, 1973^88^ Reker, 1991^90^ Silverman, 2007^98^ Solli, 2003^106^ Solli, 2008^108^ |
| **Feature** | **Impact** | **Approaches taken** | **Papers** |
| **Short length of stay** | |  |  |
| Access to only a few or even a single session | Only small clinical gains may be made Less time to work through psychotherapeutic issues Magnification of transference and counter-transference Therapeutic alliance must be built rapidly but may take time to develop. Little time for group cohesion to develop. Patient needs time to stabilise. | Analytical approaches are adapted or dropped for more behavioural, educational or supportive approaches Psychodynamic- discussions are concrete, with minimal interpretation. Transference may not be fully worked through: Focal music therapy (Storz, 2005). Here and now focus Increased frequency of sessions Open groups to engage patients earlier and increase access as soon as possible. Patients encouraged to attend as soon as behaviour allows for appropriate group interaction. Meeting patients informally outside of sessions to build rapport Therapist is more active and directive, offers more interventions and active encouragement. Therapist works flexibly and adapts session to meet needs of patients. Setting, structure and context defined at the beginning of each session with broad ground rules: Single session framework Structured music activities eg. music selection, pre-composed songs, theme based improvisation to aid accessibility of group. Therapeutic teaching not used as time too short (Priestley 1975). Goals are adjusted and prioritised to be achievable in short term– focus on coping, reduction of hospital anxiety, relaxation, stabilisation, integration, musical resources in/outside hospital, relapse prevention, interpersonal problems. Aim for continuity between services | Arnason, 1993^29^ Blake & Bishop, 1994^31^ Bonde et al. 2012^32^ Bunt, Pike & Wren, 1987^34^ Cassity & Cassity, 2006^36^ Cullen, 1993^39^ Davies & Richards, 1998^40^ Dvorkin, 2008^43^ Featherstone, 2008^47^ Frederiksen & Lindvang, 1998^49^ Gold et al., 2009^4^ Goldberg et al, 1988^53^ Goldberg, 1989^51^ Grandison, 1991^54^ Hara, 1999,^59^ Heaney, 1992^60^ Hopster, 2005^61^ Jensen, 2000^63^ Leite, 2008^65^ Moe, Roesen & Raben, 2002^73^ Morgan et al., 2011^74^ Mössler et al., 2011^76^ Moura-Costa & Negreiros Vena, 2011^77^ Murphy, 1992^79^ Odell-Miller, 1986^82^ 1992^12^ Priestley, 1975;^86^ Shultis, 1999^95^ Smith, 1975^105^ Solli, 2003^106^ Solli, 2006^107^ Solli, 2009^109^ Silverman, 2003^97^ Silverman & Marcionetti, 2004^96^ Silverman, 2009a^99^, 2009b^100^, 2010^101^,2011a^102^, 2011b^103^ Storz, 2005^112^ Strunck, 1986^114^ Sullivan, 2003^115^ Thomas, 2007^117^ Ulrich, Houtmans & Gold, 2007^118^ Wolfe, 1996^120^ |
| Little time to fully assess patient | Little time to meet patient for preliminary meeting Limited access to patient full history and background | Therapist is clear about aims, function and method of group. Use group as part of ongoing assessment Focus upon diagnosis and description. | Goldberg, 1989^51^ Nolan & Ierardi, 2007^80^ Strunck, 1986^114^ |
| Rapid discharge | No time to prepare for end of therapy Services rarely available to refer onto after discharge Low take-up of outpatient therapy | Use of receptive music selection session to decrease defence against communication. Use of closing section at end of session to reflect upon group. Establishment of good working relationships with multidisciplinary team. | Priestley, 1975^86^ Arnason, 1993^29^ Goldberg, 1994^52^ Metzner, 2010^71^ Murphy, 1992^79^ Talwar et al., 2006^116^ |
| Greater emphasis on psychopharmacological treatments | Less support/emphasis on group programme |  | Thomas, 2007^117^ |
| **Patient Turnover** |  |  |  |
| High turnover of patients | Unpredictable group membership Therapist has little control over group composition and size Lack of time to gain full history and risk assessment Level of group functioning can fluctuate Group may be composed of both new and returning members Difficult to obtain group cohesion Patients are often re-hospitalised | Admission/Assessment/Discharge processes combined Rapid uptake of new patients Goals set quickly Short term, achievable goals set Single session format Here and now focus Focus on strengthening resources of pt: resource-orientation Emphasis on continuity Structuring of events Activity confined to one session Increased frequency of sessions Opening and closing activities emphasised Rapport building outside of group Open groups Regular supervision and personal therapy of greater importance Co-therapist models role of member in group for new patients. | Arnason, 1993,^29^ Bonde et al., 2012^32^ Bunt et al., 1987^34^ Cassity & Cassity, 2006^36^ Davies & Richards, 1998^40^ Frederiksen & Lindvang, 1998^49^ Goldberg, McNiel & Binder, 1988^54^ Goldberg, 1989^51^ Goldberg, 1994^52^ Hara, 1999^59^ Hudson-Smith, 1991^62^ Leite, 2008^65^ Lindvang, 2005^67^ Lindvang & Frederiksen, 2008^66^ Lund, 2008^68^ Maler, von Wietersheim, Schurbohm, & Nagel, 1994^69^ Moss, 1999^75^ Mössler et al., 2011^76^ Murphy, 1992^79^ Odell-Miller, 1992^12^ Priestley, 1975^86^ Shultis, 1999^95^ Silverman, 2009a^99^ Silverman ,2011a^102^ Solli, 2003^106^ Solli, 2006^107^ Solli, 2009^109^ Sloboda, 2008^104^ Thomas, 2007^117^ |
| **Multi-disciplinary team** | |  |  |
| Lack of communication | Splitting of teams Lack of support in referrals, limited attendance Coordination of therapy and attendance Scheduling of patient appointments at conflicting times | Staff co-therapist used Therapist makes efforts to learn about patient-staff relationships and is informed by patient’s interaction with team: Triadic structures (Metzner, 2003,2010) Clear referral criteria Music therapist is present on wards as much as possible Significant events fed back to multidisciplinary team immediately after group. | Clemencic-Jones, 1998^38^ Fenwick, 1970^48^ Gibson et al., 2008^50^ Goldberg, 1994^52^ Metzner, 2003^70^ Metzner, 2010^71^ Odell-Miller, 1992^12^ Priestley, 1975^86^ Silverman, 2003^97^ Silverman, 2009b^100^ Solli, 2009^109^ Vogt-Schaeffer, 1991^120^ |
| Lack of understanding due to differing models/frameworks | Communication difficulties Rivalry Staff see music therapy as occupation, activity or distraction rather than psychotherapeutic intervention Staff uncertainty in when to refer Limited referrals, attendance | Therapist strives to communicate work in terms of meaning for diagnosis Opportunities offered for staff to be involved in or observe sessions Music therapist consistently reminds staff of the purpose of music therapy. Dedicated member of staff on ward to assist in music therapy session. Language is developed to communicate with multidisciplinary team – strive for collaboration and flexibility whilst acknowledging boundaries and distinct roles. | Bonde et al., 2012^32^ Goldberg et al, 1988^53^ Grandison, 1991^54^ Hannibal, 2002^57^ Hannibal et al., 2012^58^ Lindvang & Frederiksen, 2008^66^ Maler et al., 1994^69^ Moss, 1999^75^ Murphy, 1991^78^ Pavlicevic, 1987^85^ Reker, 1991^90^ Rowland & Reed, 2011^91^ Sloboda, 2008^104^ Vogt-Schaeffer, 1991^120^ |
| Lack of staff time | Limited contact with music therapist, lack of communication Difficulty finding time to meet Lack of time to do full team assessments Staff unavailable to escort patients | Handover from ward staff prior to group. Protected time for ward staff to liaise with therapists Regular clear communication with staff. Regular reminders of music therapy and who it is for. Strive for good working relationship with team. Music therapist sees patient on ward to maintain continuity | Davies & Richards, 1998^40^ Gibson et al., 2008^50^ Lindvang & Frederiksen, 2008^66^ Strehlow & Piegler, 2011^113^ |
| Lack of staff support | Limited referrals, attendance | Opportunities offered for staff to be involved in or observe sessions; Hospital wide music clubs/ensembles/performances Education of staff re: music therapy Mandatory attendance of groups to foster value of group | Bunt, Pike & Wren, 1987^34^ De Backer, 2006^10^ Fenwick, 1970^48^ Frederiksen & Lindvang, 1998^66^ Moss, 1999^75^ Murphy, 1992^79^ Priestley, 1975^86^ Ragland, 1973^89^ Rowland & Reed, 2011^91^ |
| Music therapist has minimal involvement in team meetings and ward rounds, part-time posts | Visibility and acknowledgement of music therapy in group programme structure Availability to liaise with other staff |  | Arnason, 1993^29^ Goldberg, 1994^52^ Moss, 1999^75^ Silverman, 2007^98^ |
| **Diversity of client group** | |  |  |
| Range of diagnoses, levels of experience. | Difficult to perform standardised assessments Differing musical preferences Fewer women attend group Diverse range of needs | Flexibility Specific groups for different levels of functioning/need Graded activities from low 🡪 high functioning over session Range of music interventions Therapist accepts and meets patient at their preferred level of music making as a musician. Co-therapists to meet individual needs in the group. Focus upon strengths and togetherness | Arnason, 1993^29^ Braswell et al, 1986^33^ Dye, 1993^44^ Fenwick, 1970^48^ Grandison, 1991^54^ Hara, 1999^59^ Hannibal, 2005^57^ Jensen, 2000^63^  Leite, 2008^65^ Lindvang & Frederiksen, 2008^66^ Moe 2002^72^ Moss, 1989^75^ Mössler et al., 2011^76^ Rowland & Reed, 2011^91^ Solli, 2003^106^2006^107^2009^109^ Thomas, 2007^118^ |
| Different levels of functioning | Formulation of goals Often very low functioning Group may only function to lowest member in group Higher functioning patients devote energy to supporting lower functioning patients May fluctuate over admission | Goals must be achievable: re-educative goals rather than insight- general goals of socialisation, attention span, tolerance. Use music interventions flexibly Use structure and supportive techniques to assist lower functioning patients Therapist adapts methods to functioning level of patient. Separate groups for high/low function. Low function – greater therapist activity and direction, with musical structure, and nonverbal modelling. High function – more abstract, greater freedom of expression and reflection. Graded involvement from low level to higher level groups- require more active therapist participation at beginning. Single session structured to provide higher functioning activities towards end of group. Focused groups for specific needs. Individual music therapy offered if unable to participate in group. Therapist works with different levels –ward as community, small group, individual, patients in community. Singing and ensemble groups used in open groups to build cohesion, rhythmic exercise, live music reception Composition used with higher functioning patients. Meet with patient prior to session to assess current level of functioning. | Arnason, 1993^29^ Bonde et al, 2012^32^ Bunt, Pike & Wren, 1987^34^ Cassity & Cassity, 1994^35^ Clemencic-Jones, 1998^38^ Eyre, 2011^46^ Frederiksen & Lindvang, 1998^66^ Gold et al., 2009^4^ Goldberg et al., 1988^53^ Goldberg, 1989^52^ Leite, 2008^65^ Lund, 2008^68^ Mössler et al., 2011^76^ Murphy, 1991^78^,1992^79^ Ragland, 1973^88^ Sekeles, 1999^94^ Shultis, 1999^95^ Solli, 2003^106^2006^107^2008^108^ 2009^109^ Sullivan, 2003^115^ Thomas, 2007^117^ Ulrich et al, 2007^118^ Wolfe, 1996^120^ |
| Patient previous musical experiences | Anxieties of being unmusical/getting it right/knowing what to do Unfamiliarity with active music making Previous negative experiences of music therapy or music making- tend to choose adult instruments Previous musical experience can assist in making higher levels of musical contact Skill and fluency valued more by patients than therapeutic aims Fear of breaking instruments Conflict between members with/without musical experience Patients attend with musical expectations rather than wish to work on problems. | Therapist should respond to and acknowledge anxieties held by patient- therapist takes role of educator to familiarise patients to music therapy and set rules and limits. Therapist is supportive rather than goal oriented until familiar with music therapy . Therapist should acknowledge musical background of patient as a resource; pre-group interview to assess musical preferences. Therapist works with group to find music that they can play together Use of single chord tuning for easy access on guitar. Musical structure and given rules provided to encourage patients to find their own musical language. Limited range of instruments to begin, clear instructions. Wide range of music and styles needed by therapist. Musicians encouraged to proactively maintain their skills. | Ansdell & Meehan, 2010^28^ Bunt, Pike & Wren, 1987^34^ Cassity, 1976^37^ Davies & Richards, 1999^40^ De Backer & Van Camp (2003)^41^ De Backer, 2006^10^ Dvorkin, 2008^43^ Mössler et al., 2011^76^ Odell-Miller, Hughes & Westacott, 2006^84^ Priestley, 1975^86^ Pavlicevic, 1987^85^ Reker, 1991^90^ Solli, 2003^106^, 2006^107^,2009^109^ Strunck, 1986^114^ Wolfe, 1996^120^ |
| **Symptom acuity and severity** | |  |  |
| Severity of symptoms | Ability to access or participate in music therapy may take time before able to interact in morning ability to attend session Level of medication impacts upon responses Verbal reflection is difficult- often referred as unable to access verbal psychotherapy Patients may be seen only in terms of pathology Diagnosis may be unclear May have dangerous behaviour May fluctuate over course of hospitalisation | Therapist is careful to distinguish between symptoms, patient personality and therapist’s own reactions. Limited interpretation. Therapist provides music therapy at bedside or on ward. Safety taken into account- staff handover, location of group, instruments provided. Provision of taped music (precomposed or of group) if unable to attend Therapist waits for patient to stabilise on medication, and is flexible in meeting arrangements. Therapist focuses upon musical interaction and is patient led Therapist uses end of session to ground and reorientate patients back to hospital | Abs, 1983^27^ Arnason, 1993^29^ Eyre, 2011^46^ Featherstone, 2008^47^ Frederiksen & Lindvang, 1998^66^ Goldberg, 1994^52^ Haase & Reinhardt, 2011^55^ Hannibal, 2005^57^ Hudson-Smith, 1991^62^ Leite, 2008^65^ Metzner, 2003^70^ Mossler et al., 2011^76^ Murphy, 1991^78^ Pavlicevic, 1987^85^ Priestley, 1975^86^ Reker, 1991^90^ Solli, 2003^106^,2006^107^,2008^108^,2009^109^ Solli & Rolvsjord, 2009^111^ Strehlow & Piegler, 2011^113^ Sullivan, 2003^115^ Thomas, 2007^117^ |
| Demoralisation, hopelessness, low self-esteem, isolation, alienation | Disruption Attendance ‘Fixed’ sense of illness Patients may choose music that is indicative of their symptoms (eg. melancholic music in depression) | Supportive approaches favoured Focus on promoting patient resources and strengths Structuring of session Consistency of material Greater direction from therapist Clear boundaries Movement to music to activate patients. | Arnason, 1993^29^ Cassity & Cassity, 2006^37^ Cullen, 1993^39^ Davies & Richards, 1998^40^ Fenwick, 1970^48^ Goldberg,1994^52^ Hara, 1999^59^ Hudson-Smith, 1991^62^ Priestley, 1975^86^ Rolvsjord, 2010^13^ Silverman, 2007^98^ Solli, 2003,2006,2008,2009^106-9^ Solli & Rolvsjord, 2009^110^ Strunck, 1986^114^ |
| Positive symptoms of psychosis | May exacerbate symptoms May distress other group members Rigid and stereotyped play – difficult to form musical relationship with therapist. | GIM contraindicated or adapted - goals limited to short term . Concrete focus on musical interaction – greater use of musical structure and ground rules, limited interpretation. Polarisation technique with high structure (Jensen, 2002). Factors of music therapy for recovery: motivation, structure, emotional expression, social participation (Solli & Rolvsjord, 2009). Separate group offered for acutely psychotic patients or individual improvisational music therapy. Songwriting used to communicate inner experiences. | Arnason, 1993^29^ de Backer, 2006^10^ Goldberg, 1989^51^ Goldberg, 1994^52^ Leite, 2008^65^ Moe, 2002^72^ Moe et al., 2000^73^ Jensen, 2002^64^ Seitz, 2002^93^ Solli & Rolvsjord, 2009^110^ |
| Low concentration/ attention span | Patients unable to focus for long Concrete thinking | Therapist keeps music pieces short to maintain focus. Simple musical structures. Use music to organise thought- concrete musical experiences such as singing, ensemble playing and structured songwriting. | Dvorkin, 2008^43^ Nolan & Ierardi, 2007^80^ Priestley, 1975^86^ Reker, 1991^90^ Solli, 2003^106^,2006^107^ Solli & Rolvsjord, 2009^110^ |
| Lack of motivation to attend or participate | Attendance Potential for coercion | Focus on engagement of patients Acceptance of group at own level of development Structured 3 level assessment focusing upon 1. structures on given rule, 2. inter-relationship in music, 3. symbolic/referential rules. | Cullen, 1993^39^ Hannibal, 2002^56^, 2005^57^ Lindvang & Frederiksen, 2008^66^ Moura-Costa, 2011^77^ |
| Low psychological defences | Current approaches may not be appropriate | GIM process modified- shorter structured imagery, supportive music (soft tones, harmonies, predictable structure, pleasant tempo). Concurrent drawing/writing. Patients viewed as individuals within a group setting. | Goldberg, 1994^52^ Moe, 2002^72^ |
| Anxiety, insecurity and uncertainty | Need for reassurance Attendance- difficulty to stay in group for entire session Occur particularly at beginning of therapy, particularly if a new experience Therapeutic relationship is fragile | Consistency of material Clear structure Therapist is informed by counter-transference but does not interpret. Reassurance that all are ‘good enough’ to play. If unable to access group , individual music therapy or music for relaxation is offered. | Arnason, 1993^29^ Cullen, 1993^39^ Hara, 1999^59^ Hudson-Smith, 1991^62^ Leite, 2008^65^ Mössler, Fuchs, Heldal et al., 2011,^76^ Odell-Miller, 1986^82^ Reker, 1991^90^ Shultis, 1999^95^ Solli, 2003^107^, 2006^108^,2009^110^ Stige, 2011^111^ |
| High levels of anger, impulsivity and disorganisation | Disruption Safety of group Ability of patient to plan ahead Ability to stay and commit to group Ability to reflect upon behaviour Initial difficulty in playing rhythmically | Rhythmic activities to release anger and tension. Therapist makes aims of session clear, and provides clear instructions. Avante garde music listening used to evoke and promote discussion with angry patients. Movement activities to contain mania Clear boundaries- may be reinforced musically Focus on here and now. Therapist waits until in more rational state to feedback behaviour and impact on group. Behavioural contract | Goldberg, 1989^51^ Hara, 1999^59^ Jensen, 2000^63^ Leite, 2008^65^ Lindvang & Frederikson, 2008^66^ Morgan et al., 2011^74^ Murphy, 1991^78^ Moss, 1999^75^ Priestley, 1975^86^ Silverman, 2003^97^ Solli, 2006^107^ Wolfe, 2006^120^ |
| Insight into illness | Attendance and engagement Denial of need for treatment Patients not receptive to interpretative interventions Intellectualisation of music | Opportunities to reflect may aid increase in insight Encouragement of affective responses to music. Traditional psychodynamic approaches not appropriate | Arnason 1993^29^ Cullen, 1993^39^ Hara, 1999^59^ Lindvang & Frederikson, 2008^66^ Sekeles, 1999^94^ Solli, 2006^107^ |
| Limited social and communication skills | Patients have difficulty relating to one another in group. Patients have difficulty making links between music making and behaviour. May be difficult to stimulate social interaction Patient may dominate group Sensorial play in psychosis (de Backer, 2006) May be mute | Core aim of music therapy to develop social awareness and interaction Structure and eye contact to begin communication Clear boundaries. Co-therapist to be available to individual members throughout session. Therapist asks direct open-ended questions. Therapist aims to move to playing together using gradual introduction of musical melody, structure and form. Know and respect limitations of patient (de Backer, 2006) Communication with family/friends to understand patient’s musical preferences. Different groups for differing needs/levels of functioning. | Cullen, 1993^39^ Davies & Richards, 1998^40^ De Backer, 2006^10^ Eyre, 2011^46^ Goldberg, 1994^52^ Metzner, 2010^71^ Moura-Costa, 2011^77^ Murphy, 1992^79^ Priestley, 1975^86^ Silverman, 2009a^100^ Solli, 2003,2006,2008,2009^107-109^ Solli & Rolvsjord, 2009^110^ Ulrich et al., 2007^118^ |
| **Hospitalisation** | |  |  |
| Admission may be involuntary | Patients unwilling to accept treatment Patients are disempowered Patients may attempt to show adjusted behaviour or disclose feelings in hope of early discharge Patients may abscond or threaten to leave | Aim to empower patients through collaboration and recognition of patient’s own self-knowledge Team communication vital for ongoing support of patient Music therapy used as a means of encouraging patient to stay | Arnason, 1993^29^ Haase & Reinhardt, 2011^35^ Murphy, 1991^78^ Nolan, 1991^81^ Rolvsjord, 2010^13^ Solli, 2003^106^,2006^107^ |
| Impact of hospitalisation- disruptive, frightening or punitive | Focus on immediate basic needs-initial themes of sadness, isolation, anger Patients hold concrete concerns eg. accommodation/leave Focus upon discharge and hospitalisation in session themes Therapists may be viewed as a punitive authority figure Repeat admissions Uptake of outpatient appointments is rare May use music to avoid issues | Therapist provides opportunity to express feeling states in music and names them but does not interpret. Opportunities to reflect upon issues around hospitalisation or events leading up to hospitalisation within music therapy Aims to build defences and boundaries rather than direct symptom reduction- build on resources and promote hope. May need to have opportunity to relax and dissipate anxiety Past experiences with service and staff should be taken into account Therapist arranges home visit to finish therapy | Ansdell & Meehan, 2010^28^ Arnason, 1993^29^ Baumgarten & Mahns, 1986^30^ Clemencic-Jones, 1996^38^ Cullen, 1993^39^ Davies & Richards, 1998^40^ De Backer, 2006^10^ Dvorkin, 1998^43^ Eyre, 2011^46^ Goldberg et al, 1988^53^ Goldberg, 1994^52^ Hara, 1999^59^ Hopster, 2005^61^ Jensen, 2002^64^ Leite, 2008^65^ Mössler et al., 2011^76^ Nolan & Ierardi, 2007^80^ Solli, 2006^107^ Thomas, 2007^117^ Priestley 1975^86^ Reker, 1991^90^ Talwar et al., 2006^116^ Thomas, 2007^117^ Vogt-Schaeffer, 1991^119^ |
| Limited freedom | Focus on authority/resentment Coercion- may have little choice in coming to music therapy Willingness to enter into therapy cannot be assumed- difficult to build therapeutic alliance | Important to offer patients a choice within music therapy Voluntary attendance Therapist must accept some patients may not be ready or willing to accept therapy | Abs, 1983^27^ Arnason, 1993^29^ Goldberg, 1994^52^ Pavlicevic, 1987^85^ Procter, 2002^87^ Solli, 2003^106^, 2006^107^ |
| Discharge anxiety | Attempts to split team | Strive to communicate fully with team and forge strong communicative relationships with other staff Discussion of feelings regarding discharge forms part of session Aim to facilitate return to community | Hudson-Smith, 1991^62^ Silverman, 2009a^99^ Wolfe, 1996^120^ |
| **Previous patient experiences of music therapy** | |  |  |
| Patient understanding of music therapy | May have little knowledge of therapeutic process May not understand how music therapy might help mental health problems Scepticism towards another therapy | Time to provide patient with information on music therapy Care in explaining music therapy Therapist gives patient time to think about engagement and does not force participation . Preliminary meetings Therapist negotiates with patient how they will work together Structured assessment used as an introductory course to familiarise patient. | Haase & Reinhardt, 2011^35^ Hannibal, 2002^56^ Hara, 1999^59^ Mössler et al., 2011^76^ Pavlicevic, 1987^85^ Rolvjsord, 2010^13^ Stige, 2011^111^ Storz, 2005^112^ Strunck, 1986^114^ |
| Patient expectations of music therapy | Patients have musical expectations rather than therapeutic expectations May fear failure or pressure to accomplish May fear being ‘read’ in music by therapist May have idealised view of music and not wish to think about problems within this perspective Desire for harmony Desire to learn an instrument or improve musical skills If does not fit expectations 🡪 may not engage | Therapist meets prior to group to clarify expectations – use of contract. Provide an indication of how patient understands and perceives music therapy Therapist is firm, decisive and open Therapist focuses upon musical interests and resources rather than problems Care with musicians – mindful this may have contributed to breakdown Graded approach to participation to lessen anxiety | Arnason, 1993^29^ Baumgarten & Mahns, 1986^30^ De Backer, 2006^10^ Exner, 1998^45^ Fenwick, 1970^48^ Lindvang & Frederiksen, 2008^66^ Mössler et al., 2011^76^ Priestley, 1975^87^ Solli, 2003^107^, 2006^108^ Strunck, 1986^114^ Wolfe, 1996^120^ |
| Follow-on from previous hospitalisation | Patient may return at a different point in therapy process eg. more able to explore deeper issues Those who attended previously are able to engage earlier on in readmission Music therapy may have been elsewhere | Use knowledge gleaned from previous admission to inform approach | Hudson-Smith, 1991^62^ Metzner, 2010^71^ Mossler et al., 2006^76^ Reker, 1991^90^ Rolvjsord, 2010^13^ Silverman, 2009a^99^, 2009b^100^, 2011b^103^ Solli, 2008^108^ |
| **Engagement in Music Therapy** | |  |  |
| Symptom severity | Patient may feel unable to attend Difficulty engaging patients in session Patients may not be able to stay for duration, arrive late or leave early Range of needs of patients | Primary aim to assess if patient can commit to therapy- 2-3 sessions offered to come to joint decision Ward member encourages attendance Patient offered choice of groups but once choice is made, attendance is mandatory Therapist is highly active, non-directive, patient led, flexible and provides loose structure. Therapist encourages participation in whatever way is possible Therapist works with patients as they approach on ward Therapist invites each patient on ward personally to group Therapists remind patients of group and purpose at weekly ward meeting Emphasise listening is ok as well as active play Voluntary attendance- Patients free to come and go in session Treatment is individualised to meet each patient’s needs Range of activities are offered to maximise engagement and meet individual needs | Arnason, 1993^29^ Clemencic-Jones, 1998^38^ Frederiksen & Lindvang, 1998^49^ Hannibal, 2002^56^ Hudson-Smith, 1991^62^ Lindvang & Frederiksen, 2008^66^ Metzner, 2003^70^ Morgan et al., 2011^74^ Mössler et al., 2011^76^ Moura-Costa et al., 2011^77^ Murphy, 1992^79^ Pavlicevic, 1987^85^ Priestley, 1975^86^ Ragland, 1973^88^ Rolvsjord, 2010^13^ Silverman, 2009a^99^, 2010^101^ Solli 2003^106^, 2006^107^, 2009^109^ Strunck, 1986^114^ Sullivan, 2003^115^ Wolfe, 2006^120^ |
| Difficulty in beginning active music making | Patients fearful/anxious/uncertain of improvising Passive patients in group Patient feels under pressure to begin Confrontational music leads to disengagement | Therapist provides clarity of goals and purpose at beginning of each session Therapist is encouraging. Minimal demands placed on patients eg. sit and look at instrument Therapist begins by suggesting themes or means of control (eg. follow a pulse) within improvisation then gradually encourages group to take the lead. Must provide supportive structure and active (sometimes directive) guiding Well known songs used to build confidence, then improvisation is built into these Relaxation tape made to address sleep problem Therapist works with patient to explore problem and find ways of lessening pressure Alliance can be built quickly in a few sessions | Baumgarten & Mahns, 1986^30^ De Backer & Van Camp, 2003^41^ De Backer, 2006^10^ Goldberg, 1994^52^ Hannibal et al, 2012^58^ Jensen, 2000^63^, 2002^64^ Leite, 2008^65^ Lindvang & Frederiksen, 2008^66^ Ready, 2011^89^ Sekeles, 1999^94^ Silverman, 2003^97^, 2009^99^ Sloboda, 2008^104^ Solli, 2003^106^, 2006^107^, 2009^109^ Stige, 2011^111^ |
| Lack of group stability impacts upon engagement | Member of staff leaving group impacted upon engagement Fluctuating attendance due to external demands Difficult to establish rapport quickly | Therapist tries to prepare for ending, and acknowledges absence afterwards. Informal meeting with patients before group, contact with patients after session Closed groups offer greater stability Greater frequency increases opportunities to attend and build cohesion Familiarity with patient musical preferences assists in building rapport | Baumgarten & Mahns, 1986^30^ Featherstone, 2008^47^ Moe, 2002^73^ Silverman, 2009a^99^, 2011a^102^ Thomas, 2007^117^ |
| Patient may not be ready to access groups | Patient is too disruptive or anxious for group Schizophrenia, female gender and less than 20 sessions associated with drop-out from treatment Modified GIM requires a level of trust and relaxation | Individual music therapy offered | Blake & Bishop, 1994^31^ Haase & Reinhardt, 2011^55^ Hannibal, 2005^57^ Solli, 2009^109^ |
| Patient unwilling to attend | Resistant to music therapy Patient presents negative comments to group or is disruptive Patient may be ambivalent about returning – wish to be discharged Patient may be coerced or attend to ‘play the system’ May reject music therapy but return at a later point | Therapist accepts and supports all musical expressions and level of group development. Uses resistance to build relationships and allow for discussion of negative feelings. Therapist provides clear temporal boundaries Freedom and flexibility offered within session to attend/participate or not Use of musical boundaries rather than verbal boundaries. Token economy system as part of overall hospital framework Rely on staff support and encouragement to attend | Arnason, 1993^29^ Cullen, 1993^39^ Grandison, 1991^54^ Hara, 1999^59^ Jensen, 2000^63^ Moura-Costa et al, 2011^77^ Nolan, 1991^81^ Procter, 2002^87^ Silverman & Marcionetti, 2004^96^ Solli, 2003^106^, 2006^107^, 2009^109^ Strunck, 1986^114^ |
